# Is Covid‐19 changing sustainable consumer behavior? A survey of Italian consumers

**DOI:** 10.1002/sd.2322

**Published:** 2022-04-27

**Authors:** Rosa Maria Dangelico, Valerio Schiaroli, Luca Fraccascia

**Affiliations:** ^1^ Department of Mechanics, Mathematics, and Management Polytechnic University of Bari Bari Italy; ^2^ Department of Computer, Control, and Management Engineering “Antonio Ruberti” Sapienza University of Rome Rome Italy; ^3^ Department of Mechanical and Aerospace Engineering Sapienza University of Rome Rome Italy; ^4^ Department of Industrial Engineering and Business Information Systems University of Twente Enschede The Netherlands

**Keywords:** consumer dynamics, Covid‐19, green consumer behavior, green products, sustainability, sustainable consumer behavior, sustainable products

## Abstract

Since the beginning of 2020, the world has been hit by the SARS‐CoV‐2 virus that causes Covid‐19. To hamper its spread, policymakers of many countries have put in place strong countermeasures, including lockdowns, that have led to significant changes in people's lifestyles and daily routines. This article aims at assessing the changes caused by Covid‐19 in sustainable consumer behavior under multiple perspectives, contributing to advance knowledge at the intersection between consumer dynamics and sustainable consumer behavior literature. A survey was conducted on 1.535 Italian consumers between December 2020 and February 2021. Respondents were asked to assess the extent to which their consumption behavior—purchase frequency, willingness to pay a premium price, sense of moral duty to purchase, social influence to purchase—related to several categories of sustainable products changed due to the pandemic, as well as the extent to which the pandemic impacted on many other aspects, including their environmental awareness, concern, and habits. Results show that Covid‐19 generated relevant changes. Consumers have increased their purchase frequency and willingness to pay for sustainable products, show growing attention to environmental issues, and behave more sustainably. Further, the extent of change is strongly affected by socio‐demographic variables, such as gender, age, income, and education. For instance, women reported a higher shift towards sustainable consumption and behavior than men. Understanding these changes is important to guide marketers and policymakers to respond promptly and effectively to them and to leverage on them to foster a transition towards a more sustainable society.

## 1 INTRODUCTION

Since the early stage of 2020, humanity has started to be threatened by the SARS‐CoV‐2 virus, which causes Covid‐19. Its contagiousness, its high mortality rate, especially for the elderly, and the need for hospitalization and intensive care for the most serious cases led policymakers of many countries to take several countermeasures: social distancing, wearing protection masks, restrictions that involve non‐essential activities (e.g., restaurants and non‐grocery shops), partial or total lockdowns, among others.

Together with the fear of the new virus, which has caused severe impacts on the global economy (Brewer & Sebby, 2021), these countermeasures have led to significant changes in people's lifestyles and daily routines (Kirk & Rifkin, 2020; Zwanka & Buff, 2021). Many studies in the literature have been conducted to investigate how and the extent to which consumer habits and behavior can be affected by particular (unexpected) events, such as natural disasters, for example, hurricanes (Sneath et al., 2009), earthquakes (Forbes, 2017), and terroristic attacks (Baumert et al., 2020; Herzenstein et al., 2015). In line with this literature, research on the impact of Covid‐19 on consumer behaviors has been rapidly growing. This topic has been analyzed from different perspectives, such as consumers' food consumption habits (Coulthard et al., 2021; Di Renzo et al., 2020; Murphy et al., 2021), shopping behavior (Moon et al., 2021; Safara, 2020; Wang et al., 2020), mobility patterns (Anke et al., 2021), physical activity (Martínez‐de‐Quel et al., 2021), and tourism (Miao et al., 2021). It has been highlighted that consumers constantly change their behavior in response to policies developed by governments to address the health emergency (Yang et al., 2021), suggesting the relevance of studying the impact of the pandemic on consumer behavior. Indeed, understanding changes in consumer behavior is of great importance to guide marketers and policymakers to respond promptly and effectively to these changes (Kirk & Rifkin, 2020).

Despite the literature on the topic has been growing and the phenomenon has been studied from different points of view, research on the influence of Covid‐19 on consumers' behavior is still at a nascent stage (Qi et al., 2020) and is an urgent issue (Borsellino et al., 2020). Further, the literature recognizes the need to investigate the impact of Covid‐19 on sustainability from multiple perspectives, including how and the extent to which the pandemic affected consumers' environmental concerns and sustainable consumer behavior (Jian et al., 2020; Sarkis et al., 2020), defined as consumer behavior motivated by social and/or environmental considerations (Gilal et al., 2020; Khan et al., 2020; Luchs & Mooradian, 2012; Rasool et al., 2021; Young et al., 2010). Deepening our knowledge on this topic would be very important to understand whether and how companies and policymakers can leverage on disasters to foster the transition towards a more sustainable society.

Thus, this study contributes to the literature on the influence of disasters on consumer behavior with specific regard to sustainable behavior. Specifically, this article assesses the extent of changes in sustainable consumer behavior caused by the Covid‐19 pandemic in Italy under multiple perspectives (i.e., awareness, concern, social influence, moral duty, willingness to pay, purchase frequency and behavior, pro‐environmental behaviors and transportation modes). Further, the change in consumer behavior for different categories of sustainable products is analyzed. Specifically, six categories are considered: organic and eco‐sustainable products (as contributing to the environmental dimension of sustainability), fair‐trade products (as taking into account the social and economic dimensions), made in Italy and local products (as positively contributing to all three dimensions ‐ being produced in Italy or locally, there is a reduced environmental impact due to transportation as well as a positive impact on Italian/local economy and employment levels), and products sold by neighborhood stores (as mainly positively contributing to the social and economic dimensions, due to their impact on the local economy and employment levels). These categories have been chosen since they reflect the three different dimensions of sustainability.

To measure the extent of changes in consumer behavior, a questionnaire was developed and administered to a sample of 1.535 Italian consumers. Specifically, we asked if the pandemic has increased consumer awareness (related to environmental problems, the relevance of social and political cooperation, and the impact of individual behaviors and purchase choices on the environment, society, and economy) and consumer concern (related to infectious disease and several environmental problems). Further, we asked if the pandemic has increased consumer sense of moral duty and perceived social influence to purchase different categories of sustainable products. Then, we asked if the pandemic has increased or decreased consumer purchase frequency and willingness to pay for these products and if the pandemic has increased or decreased several consumer behaviors and habits (i.e., purchase behaviors, pro‐environmental behaviors, and transportation modes). Differences among the answers provided by groups with different socio‐demographic characteristics (i.e., gender, age, income, and education) have been investigated and interesting results have emerged.

The article is organized as follows. In the next section, a review of the literature on consumer dynamics and the impact of the Covid‐19 pandemic on consumer behavior is provided. In section three, we explain the methodology used and describe the characteristics of the sample. In section four, we describe and discuss the results. Finally, in the last two sections, we report the implications and limitations of the study and provide insights for future research.

## 2 LITERATURE REVIEW

According to Zhang and Chang (2021), consumer dynamics can be defined as “temporal changes in consumer attitudes and behaviors.” Over time, the environment where consumers interact might change driven by economic, social, and technological transformations, resulting in a dynamic process able to affect consumer perceptions and behaviors. Nevertheless, unexpected events can affect consumer habits and behaviors. For instance, Baumert et al. (2020) and Herzenstein et al. (2015) found that terrorist threats specifically alter the preferences and buying behavior of direct victims in the short period. Several studies in the literature address how and the extent to which natural disasters can impact consumer behavior. For instance, Sneath et al. (2009) highlighted that the stressful situation due to the Katrina hurricane fed compulsive or impulsive buying behaviors. Forbes (2017) found that, just after the Christchurch earthquake, consumers increased the amounts of utilitarian products bought while purchasing lower amounts of hedonic and harmful products. Mainardes et al. (2021) found that environmental tragedies are able to enhance the consumers' awareness of the human impact on the environment, which drives more sustainable consumption habits.

The Covid‐19 pandemic is a global disruptive event that has smashed our lives. A growing number of studies are focusing on the exploration of the Covid‐19 pandemic effects on consumer behaviors from different perspectives. Some studies have assessed the changes in food consumption habits due to the Covid‐19 pandemic in several countries, such as Italy (Di Renzo et al., 2020; Vittuari et al., 2021), United Kingdom (Coulthard et al., 2021), China (Li et al., 2021), Bosnia and Herzegovina (Ben Hassen et al., 2021), United States, Ireland, and New Zealand (Murphy et al., 2021). Jribi et al. (2020) and Pappalardo et al. (2020) found that Covid‐19 has reduced consumers' food wastes and has increased the positive attitudes of consumers to prevent food wastage. Similarly, Burlea‐Schiopoiu et al. (2021) and Severo et al. (2021) found that the Covid‐19 pandemic has driven consumers towards food waste reduction behaviors and has increased the awareness of the environmental impact of food waste. Other studies analyzed changes in shopping behavior. For instance, Moon et al. (2020) and Safara (2020) found an increase in online shopping patterns at the expense of buying in physical stores. Several studies (e.g., Ben Hassen et al., 2021; Schmidt et al., 2021; Wang et al., 2020) found that consumers have reduced the shopping frequency and the time spent in physical stores while increasing the purchased quantity per shopping trip. Other studies found an increase in purchased quantities of necessities products (Di Crosta et al., 2021) and non‐perishable food and hygiene products (Schmidt et al., 2021) compared to before the Covid‐19 pandemic. Sun et al. (2021) and Qi et al. (2020) found that the Covid‐19 pandemic has driven consumers towards buying sustainable products and that consumers now pay more attention to the environment and the society. The increase in the environmental concern due to the Covid‐19 pandemic is also reported by Jian et al. (2020) and Mehta et al. (2020).

According to the literature, the Covid‐19 pandemic has also affected mobility patterns. Several studies (e.g., Anke et al., 2021; Wang et al., 2021; Xiong et al., 2021) reported large shifts in consumer mobility behaviors, characterized by drastic reductions in the use of public transport and a relevant increase in active mobility and car usage. König and Dreßler (2021) found that consumers living in rural communities reduced the use of busses and cars and increased the use of bikes. Miao et al. (2021) studied the effect of the pandemic on tourism, finding that people prefer local trips and try to avoid congested areas. Similarly, Vaishar and Šťastná (2022) found that Covid‐19 shifted consumer preferences from urban to rural tourism.

Concerning the Italian context, the research conducted on the impact of the Covid‐19 pandemic on consumer behavior is limited. In this regard, an increase in the consumption of farmers' or organic food (Di Renzo et al., 2020) and certified sustainable food products (Castellini et al., 2021) is reported. Peluso et al. (2021) found that consumers have increased their spending on environmentally sustainable products by nearly 10%. Furthermore, consumers have reduced the amount of food waste produced, mainly thanks to better household food management, being engaged in more home cooking, and the increased role of health concerns in food choices (Amicarelli et al., 2021; Borsellino et al., 2020; Cai et al., 2021; Principato et al., 2020; Rodgers et al., 2021). Regarding the shopping process, it was found that consumers reduced the shopping frequency at physical stores, buying larger quantities at a time (Degli Esposti et al., 2021; Vittuari et al., 2021), and increased the frequency of purchasing online (Alaimo et al., 2020). Further, consumers reduced the frequency of purchase of certain products, such as clothing, beauty and body care products, and apparel, while they have increased the frequency of purchase of entertainment products and services, such as books, movies, series, and video games (Degli Esposti et al., 2021). Regarding the lifestyle, Di Renzo et al. (2020) highlighted a slight increase in physical activity and a slight reduction in smoking. Cai et al. (2021) found that Italian people's mobility has decreased during the Covid‐19 pandemic, especially in large cities. In this regard, Degli Esposti et al. (2021) highlighted that consumers have reduced the use of public transport, since they prefer to avoid contact with strangers, as well as they are less likely to use car‐sharing and other forms of collaborative consumption (bike, scooter sharing, jump scooter sharing).

This review of the literature highlighted that the most relevant areas of consumer dynamics in the context of Covid‐19 for sustainable consumer behavior are the following: (a) consumers might be more prone to adopt pro‐environmental behaviors (e.g., behaviors able to reduce food wastage) thanks to an increased awareness of the human impact on the environment; (b) consumers might be more willing to purchase sustainable products (e.g., organic food) because they now pay more attention to the environment and society when making purchasing choices; and (c) consumers might be less willing to be involved in activities that may imply physical contact with other people (e.g., using public transport or other forms of shared mobility, shopping in large or physical stores) preferring to conduct alternative behaviors (e.g., using private cars, walking, purchasing products online).

Nevertheless, existing studies have focused each on single aspects of habits and lifestyle and a more comprehensive analysis, involving multiple aspects simultaneously, is lacking. In particular, there is the need to further investigate whether and the extent to which the current pandemic is inspiring pro‐environmental attitudes (Peluso et al., 2021), as well as to better understand the impact of the Covid‐19 pandemic on environmental concerns and sustainable consumer behavior (Jian et al., 2020; Sarkis et al., 2020). Moreover, as many of these studies highlighted that the impacts of the Covid‐19 pandemic on consumer behavior might be different according to several consumers' socio‐demographic characteristics ‐ such as age, gender, income, and education (Dhir et al., 2021; Li et al., 2021; Peluso et al., 2021; Rodgers et al., 2021; Severo et al., 2021; Vittuari et al., 2021; Xiong et al., 2021) ‐ there is the need to consider this issue in the analysis.

Based on this review of the literature, we identified the topics to be covered in our study.

## 3 METHODOLOGY

This section is divided into three subsections. Section 3.1 describes the questionnaire used to collect data. Section 3.2 describes the procedure of data collection and the characteristics of the respondents. Finally, Section 3.3 introduces the data analysis conducted.

### 3.1 The questionnaire

A structured questionnaire was developed to collect data for this research. Most of the scales used are based on existing scales, adapted to capture the changes due to the Covid‐19 pandemic and/or by taking into account specific categories of sustainable products. Some new items have also been added to more deeply cover the investigated topics. The questionnaire is composed of three sections. All the questions and their sources are reported in Table A1 of the Appendix. The first section included five questions. A five‐point Likert‐type scale was used to measure how much the Covid‐19 pandemic increased (from “not at all” to “extremely”) consumers' levels of awareness, concern, and moral duty, as well as social influence. Specifically, the first two questions, Q1 and Q2, investigated the changes in respondents' level of awareness and concern about different types of environmental and social problems. Q1 evaluated, through six items, the impact of Covid‐19 on the increase of respondents' awareness on the effect of human activities on environmental degradation, as well as on the relevance of cooperation among different countries, social actors, and political groups to solve complex problems (two items from BCG, 2020, four items self‐developed). Q2 investigated, through seven items, the extent to which, due to Covid‐19, respondents are more concerned of infectious diseases and several environmental problems (pollution, climate change, unsustainable management of natural resources, natural habitat destruction, biodiversity loss, and disposal of unsorted waste) (adapted from BCG, 2020). Q3 evaluated, through seven items, the influence of Covid‐19 on the increase of respondents' awareness about the impact of their purchasing decisions on the natural environment, society, and economy (adapted from Kang et al., 2013). Q4 measured, through six items, the changes due to Covid‐19 in respondents' sense of moral duty to purchase different product categories (sold by neighborhood stores, made in italy, local, fair‐trade, organic, eco‐sustainable^1^) (adapted from Beldad & Hegner, 2018). Q5 evaluated, through six items, the extent to which, due to Covid‐19, people important for the respondent believe it is right to buy more than before the previously mentioned categories of products (adapted from Alzubaidi et al., 2020).

The second section included five questions. A five‐point Likert‐type scale was used to measure the change, due to the Covid‐19 pandemic, in the frequency of different types of actions (from “I do it a lot less often” to “I do it a lot more often”) for questions Q6, Q7, Q8, and Q9. Specifically, Q6 investigated, through six items, how much the frequency of purchase of different product categories (sold by neighborhood stores, made in Italy, local, fair‐trade, organic, eco‐sustainable) changed due to the pandemic (three items from BCG, 2020, three items self‐developed). Q7 evaluated, through six items, how much the frequency of carrying out several purchase behaviors has changed due to the pandemic (two items adapted from BCG, 2020, four items self‐developed). Q8 measured, through four items, how much the frequency of different pro‐environmental behaviors changed due to the pandemic (adapted from BCG, 2020). Q9 evaluated, through five items, how much the frequency of using different means of transport changed due to the pandemic (one item from BCG, 2020, four items self‐developed). Q10, through five items, investigated the changes due to Covid‐19 in respondents' willingness to pay a premium price for five product categories (Made in Italy, Local, Fair‐trade, Organic, and Eco‐sustainable)^2^ (adapted from Moser, 2015). For Q10, a five‐point Likert‐type scale was used (from “greatly decreased” to “greatly increased”).^3^

Finally, the third section included questions on socio‐demographic characteristics of respondents (gender, age, education, net monthly household income)^4^ (Barbarossa & De Pelsmacker, 2016; Dangelico et al., 2021; Magnier et al., 2019).

### 3.2 Procedure and sample

The developed questionnaire was pre‐tested with a sample of 40 people to check for the clarity of questions. As a result, small changes in the wording of some sentences were done to improve their clarity.

The questionnaire was administered through the Qualtrics survey platform. The survey was distributed to people living in Italy, between December 2020 and February 2021, through multimedia channels (e.g., social networks and instant messaging clients), selecting a convenience sample through the non‐probabilistic snowball method (Noy, 2008). Accordingly, participants were asked to respond to the questionnaire and share the survey as much as possible. The snowball sampling method is widely applied in the literature (e.g., Agadayi et al., 2021; Kooli et al., 2019; Tamrin et al., 2021), especially by studies on consumer behavior (Alaimo et al., 2020; Di Renzo et al., 2020; Jian et al., 2020; Peluso et al., 2021).

In our survey, all the questions were mandatory: without having provided an answer to one question, respondents were disallowed to continue to the next questions. Hence, respondents were allowed to finally submit the questionnaire only if they had replied to all the questions. Therefore, there were no missing values in the dataset. The final sample is made of 1.535 respondents. The socio‐demographic characteristics of the sample are reported in Table 1.

**TABLE 1:** Socio‐demographic characteristics of the sample

Variable	Group	Frequency	Percentage (%)
Gender	Male	668	43.52
	Female	867	56.48
Age	18–24	347	22.61
	25–34	480	31.27
	35–44	215	14.01
	45–54	235	15.31
	55–65	198	12.90
	> 65	60	3.91
Education	Middle school or lower	84	5.47
	High school	563	36.68
	Bachelor's master's degree	705	45.93
	Postgraduate course or PhD	183	11.92
Net monthly household income (euros)	<1.500	258	16.81
	1.500–3.000	789	51.40
	3.001–4.500	325	21.17
	>4.500	163	10.62
Total		1535	100

### 3.3 Data analysis

The IBM SPSS v26.0 software was used to analyze the data. First, the data distribution was checked through the Shapiro–Wilk test, revealing that the data variables were not normally distributed. Then, for each question, the nonparametric Kruskal–Wallis test was performed, in order to highlight whether statistically significant differences among the different socio‐demographic groups exist. Finally, a post hoc test comparing pairwise via Dunn procedure with Bonferroni adjustment was conducted, aimed at highlighting among which groups these significant differences exist. Accordingly, the differences in the consumer behavior of each socio‐demographic group can be highlighted. A statistical significance level of 0.05 was chosen for all the tests used to analyze data.

**FIGURE 1:**
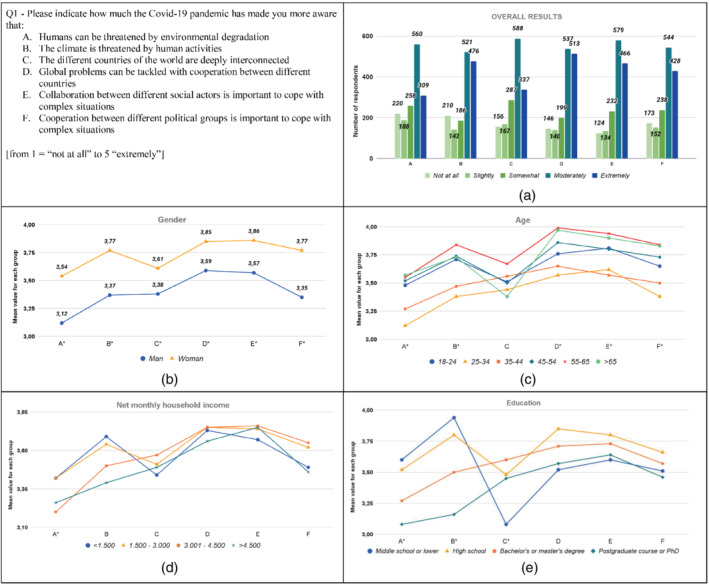
Increase, due to the Covid‐19 pandemic, in the level of awareness of environmental problems and relevance of cooperation to solve complex/global problems. (a) Overall results; (b) gender; (c) age; (d) household income; (e) education. * means that significant differences exist among groups [Colour figure can be viewed at wileyonlinelibrary.com]

**FIGURE 2:**
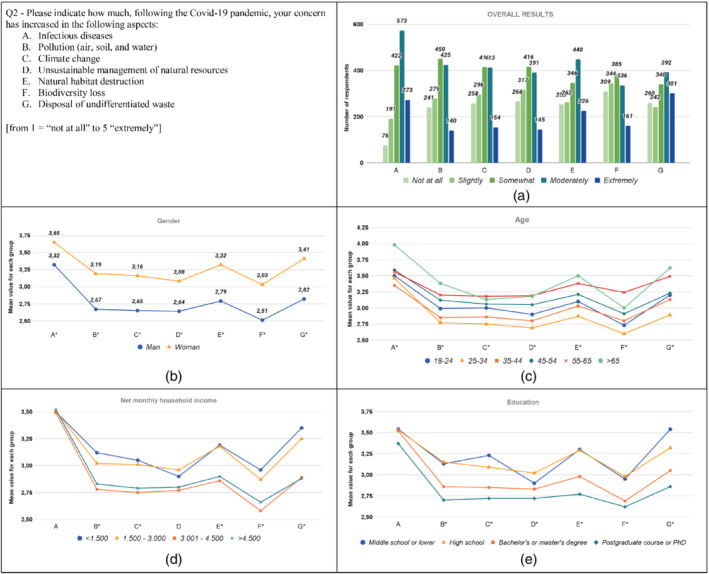
Increase in the level of concern about environmental problems and infectious disease due to the Covid‐19 pandemic. (a) Overall results; (b) gender; (c) age; (d) household income; (e) education. * means that significant differences exist among groups [Colour figure can be viewed at wileyonlinelibrary.com]

**FIGURE 3:**
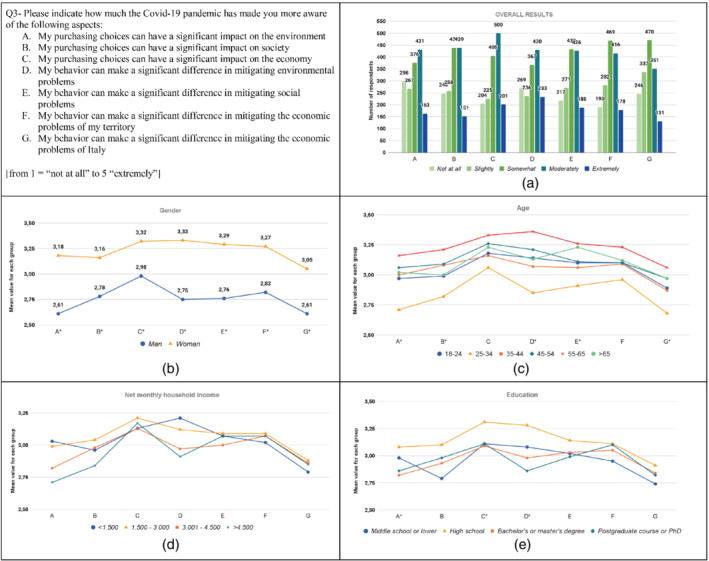
Increase in the level of awareness about the impact of consumer purchasing choices and behaviors due to the Covid‐19 pandemic. (a) Overall results; (b) gender; (c) age; (d) household income; (e) education. * means that significant differences exist among groups [Colour figure can be viewed at wileyonlinelibrary.com]

**FIGURE 4:**
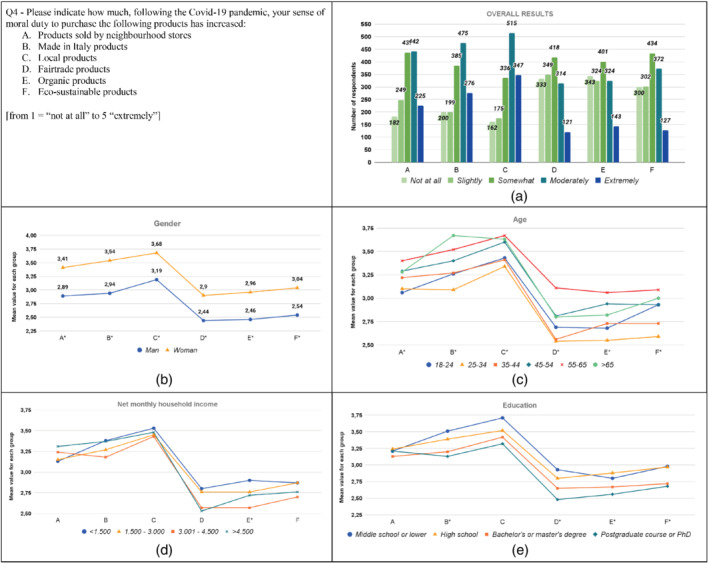
Increase in the level of moral duty to purchase specific categories of products due to the Covid‐19 pandemic. (a) Overall results; (b) gender; (c) age; (d) household income; (e) education. * means that significant differences exist among groups [Colour figure can be viewed at wileyonlinelibrary.com]

**FIGURE 5:**
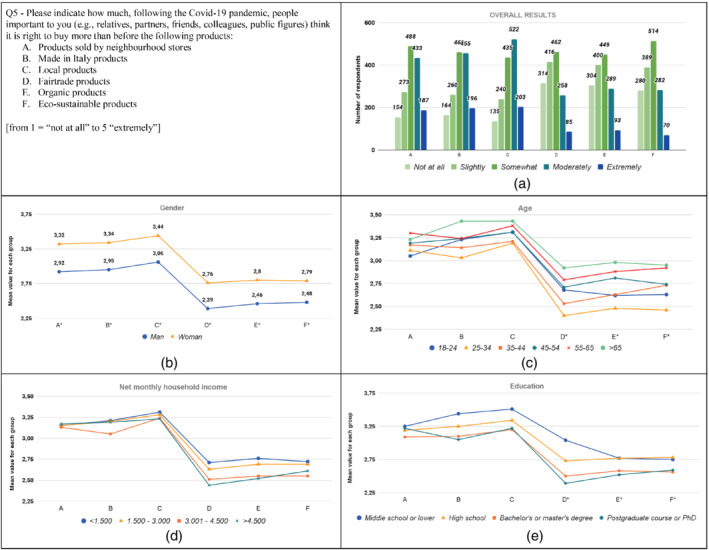
Increase in the level of social influence exerted on purchase behavior for different product categories due to the Covid‐19 pandemic. (a) Overall results; (b) gender; (c) age; (d) household income; (e) education. * means that significant differences exist among groups [Colour figure can be viewed at wileyonlinelibrary.com]

**FIGURE 6:**
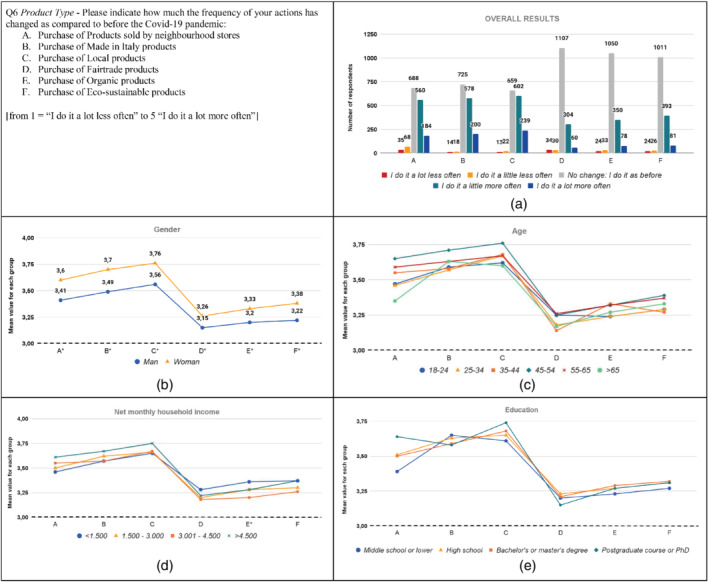
Change in the purchase frequency of sustainable products due to the Covid‐19 pandemic. (a) Overall results; (b) gender; (c) age; (d) household income; (e) education. * means that significant differences exist among groups. The dotted line represents the reference value for no change before the Covid‐19 pandemic [Colour figure can be viewed at wileyonlinelibrary.com]

**FIGURE 7:**
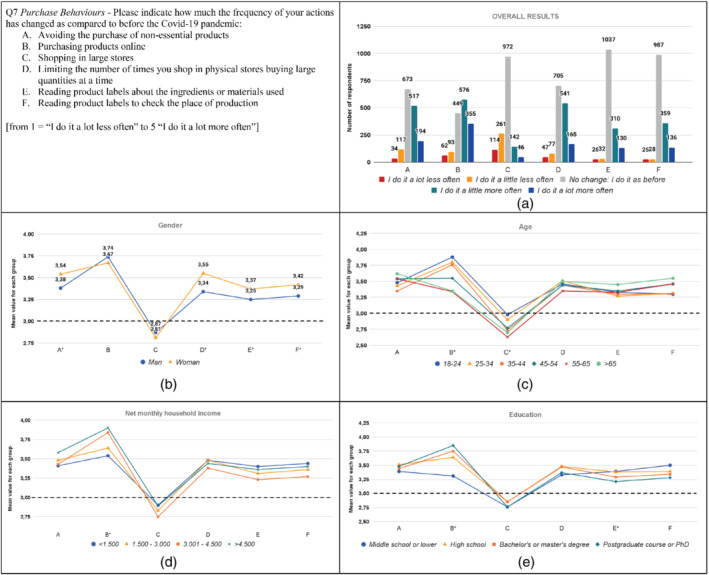
Change in purchase behaviors due to the Covid‐19 pandemic. (a) Overall results; (b) gender; (c) age; (d) household income; (e) education. * means that significant differences exist among groups. The dotted line represents the reference value for no change before the Covid‐19 pandemic [Colour figure can be viewed at wileyonlinelibrary.com]

**FIGURE 8:**
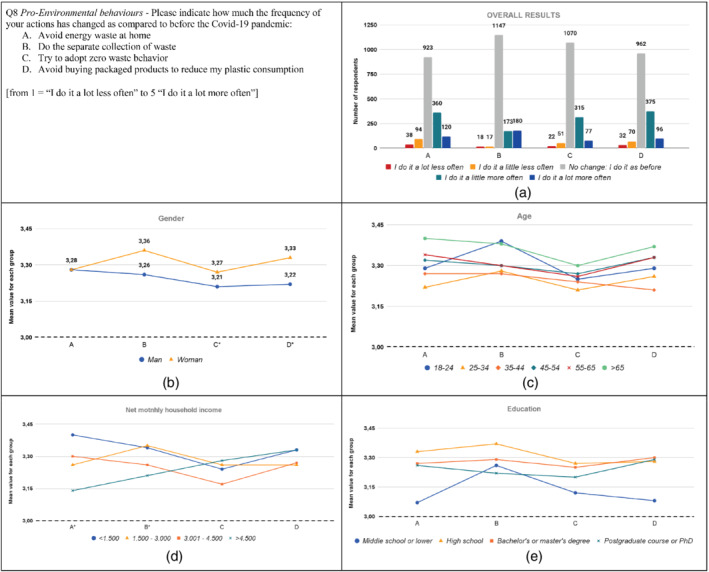
Change in pro‐environmental behaviors due to the Covid‐19 pandemic. (a) Overall results; (b) gender; (c) age; (d) household income; (e) education. * means that significant differences exist among groups. The dotted line represents the reference value for no change before the Covid‐19 pandemic [Colour figure can be viewed at wileyonlinelibrary.com]

**FIGURE 9:**
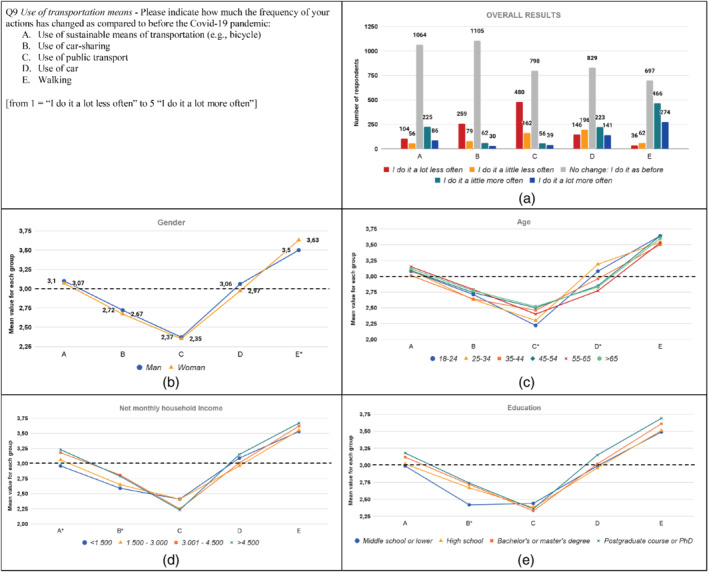
Change in the use of transportation means due to the Covid‐19 pandemic. (a) Overall results; (b) gender; (c) age; (d) household income; (e) education. * means that significant differences exist among groups. The dotted line represents the reference value for no change before the Covid‐19 pandemic [Colour figure can be viewed at wileyonlinelibrary.com]

**FIGURE 10:**
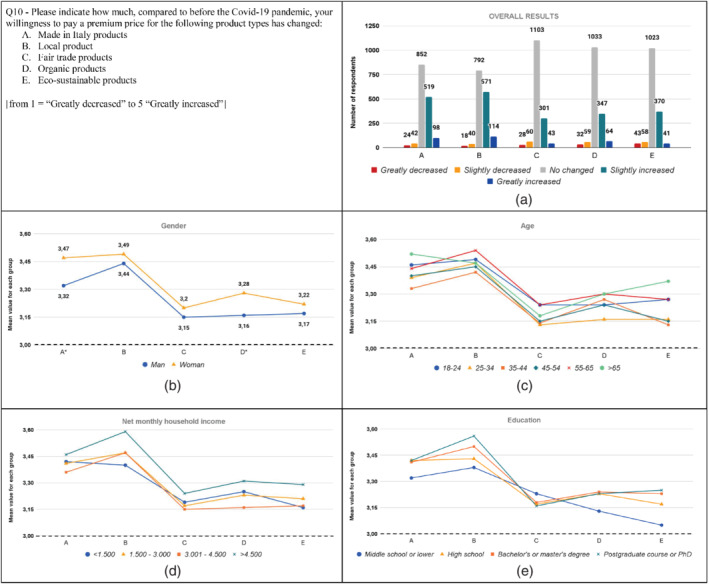
Changes in the willingness to pay a premium price for sustainable products due to the Covid‐19 pandemic. (a) Overall results; (b) gender; (c) age; (d) household income; (e) education. * means that significant differences exist among groups. The dotted line represents the reference value for no change before the Covid‐19 pandemic [Colour figure can be viewed at wileyonlinelibrary.com]

The results from the analysis are graphically displayed in Figures 1, 2, 3, 4, 5, 6, 7, 8, 9, 10. Each figure reports a specific question with its items; each item is indexed with a letter, which is used to identify it in the graphs. First, for each item of the questionnaire we showed the descriptive analysis of the whole sample, defining the responses value distribution on the five scale anchors (Figures 1, 2, 3, 4, 5, 6, 7, 8, 9, 10a). Then, to display the results of the Kruskal–Wallis tests, we created a graph for each of the four socio‐demographic categories considered (Figures 1, 2, 3, 4, 5, 6, 7, 8, 9, 10b–e). Here, for each statement, we reported the mean score for each group using lines of different colors, which allow us to highlight similarities or differences among groups. Whether statistically significant differences do exist among groups, the “*” superscript over the letter denoting the specific item is added. The reference value for no change is highlighted with a dotted line in Figures 6, 7, 8, 9, 10.

## 4 RESULTS AND DISCUSSION

In this section, results from data analysis are reported and discussed. For each question, first the results on the whole sample of responses are provided. Then, the results for different groups of respondents—defined according to socio‐demographic characteristics—are compared and statistically significant differences among groups are highlighted. Full results of statistical tests for each question are reported in Tables A2, A3, A4, and A5 of the Appendix.

### 4.1 Q1: Awareness of environmental problems and importance of cooperation to solve complex/global problems

The results show that the pandemic increased the awareness of respondents related to all the considered aspects, in particular that “Global problems can be tackled with cooperation among different countries”, “Collaboration among different social actors is important to cope with complex situations”, and “The climate is threatened by human activities” (Figure 1a). More than 60% of respondents stated a moderate or extreme increase in the level of awareness. Similar results are reported by Castellini et al. (2021), who highlighted an increase in consumers' awareness of the importance of environmental issues and animal welfare in Italy, as well as by Severo et al. (2020), who highlighted an increase in environmental awareness of Brazilian and Portuguese consumers due to the pandemic. In contrast, the study conducted of several European countries by Rousseau and Deschacht (2020) highlighted that the awareness of environmental issues was not affected.

The level of awareness increased for all the considered groups. Women report significantly higher values than men for all the statements (Figure 1b; Table A2). Statistically significant differences can be noticed also among the age groups (see Table A3), except for the statement C. Figure 1c shows that the distribution of the responses for the “25–34” and “35–44” groups is similar, and the respective values are lower than those of the other groups. On multiple statements, the increase in the awareness of the group “25–34” is significantly lower compared to groups “18–24”, “45–54”, and “55–65.” Alternatively, the increase in the awareness of the group “55–65” is significantly higher compared to groups “18–24”, “25–34”, and “35–44.” Regarding the household income, no significant differences can be noted among groups, except for the statement A (Figure 1d; Table A4). Here, the “1.500–3.000” group shows a higher increase in the level of awareness about the threat by environmental degradation than the group “3.001–4.500.” With respect to education, statistical differences among groups are found on the first three statements (Figure 1e; Table A5). Specifically, respondents whose education level is “Postgraduate course or PhD” have a lower increase in the level of awareness for environmental problems (statements A and B) compared to other groups, while respondents whose education level is “Middle school or lower” reported a lower increase in the awareness than other groups on statement C.

### 4.2 Q2: Concern about infectious disease and environmental problems

The results show that the pandemic increased the concern of respondents related to all the considered aspects (Figure 2a). Not surprisingly, the highest increase in the level of concern is about “Infectious diseases”, with around 55% of the answers on the last two anchors of the scale (i.e., “Moderately” and “Extremely”). Around 45% of the respondents stated their level of concern about “Destruction of the natural habitat” and “Disposal of undifferentiated waste” increased “Moderately” or “Extremely.” This result is consistent with Jian et al. (2020) and Severo et al. (2021), who highlighted the increase in concern for environmental issues due to Covid‐19.

The level of concern increased for all the considered groups. It can be noted that women reported significantly higher values than men for all the statements investigated (Figure 2b; Table A2). This result is in line with Abdullah et al. (2020), who stated that women are more concerned about Covid‐19 infection. Results reveal significant differences among the age groups for each statement (Figure 2c; Table A3). The increase in the level of concern for groups “25–34” and “35–44” is statistically lower than the other groups on multiple statements. These results could be due to an already high starting level for these groups that moderated the pandemic effects, but further research would be needed to investigate this issue. Alternatively, the groups “45–54”, “55–65”, and “> 65” are characterized by higher increases in levels of concern. This is in line with the study by Dhir et al. (2021), which highlighted that older consumers are more environmentally concerned than younger ones. Respondents over 65 increased the level of concern about infectious diseases statistically more than other groups. This pattern can be due to the fact that old people have higher risks associated with Covid‐19 than young people. Significant differences among *household income* groups can be noted for all the statements except for two aspects: “Infectious disease” ‐ as all the income groups report a similar increase in the level of concern about this statement ‐ and “Unsustainable management of natural resources” (Figure 2d; Table A4). Significant differences emerged among respondents, depending on whether their net monthly income is lower or higher than 3.000 euros. In this regard, the higher the income, the lower the increase in the level of concern. Regarding education (see Table A5), Figure 2e shows significant differences among groups for all the considered aspects, except for “Infectious Diseases.” Overall, the level of concern of respondents whose education level is “Postgraduate course or PhD” increased less compared to the other respondents. On the contrary, respondents whose education level is “Middle school or lower” and “High school” increased their level of concern statistically more than respondents belonging to the other groups.

### 4.3 Q3: Awareness of the impact of consumer purchasing choices and behaviors

The results show that the pandemic caused an increase in consumer awareness related to all the considered aspects (Figure 3a). Over 40% of respondents became moderately or extremely more aware that their individual purchasing choices can have a significant impact on the economy and that their behavior can make a significant difference in mitigating environmental problems. Overall, for all the other aspects, over 30% of the answers are on the last two anchors of the scale. These results are consistent with the study by Castellini et al. (2021) conducted on Italian consumers, which reported an increase in consumers' awareness of the importance of individual responsibility in society, and with the study by Sun et al. (2021) conducted on Chinese consumers, which found that the emotional response induced by the Covid‐19 pandemic has driven consumers to pay more attention to the environment and society.

The level of awareness increased for all the socio‐demographic groups. Women report significantly higher values than men for all the statements (Figure 3b; Table A2). Concerning the age groups, significant differences among the groups are found for all the statements, except for statements C and F (Figure 3c; Table A3). The “25–34” group has a significantly lower level of increase compared to the “55–65” and “45–54” groups on multiple statements. No significant differences among the household income groups are found (Figure 3d; Table A4), whereas significant differences among education groups are found for the statements A, C, and D (Figure 3e; Table A5), for which the “High school” group has significantly higher values than the “Bachelor's or master's degree” and “Postgraduate course or PhD” groups. For all the statements, the “High school” group displays the highest increase in the level of awareness.

### 4.4 Q4: Moral duty to purchase sustainable products

Results show that the pandemic increased consumers' moral duty to purchase all the considered product categories (Figure 4a). The sense of moral duty to purchase Local products moderately or extremely increased for over 50% of respondents, whereas the sense of moral duty to purchase Made in Italy products and Products sold by neighborhood stores moderately or extremely grew for over 40% of respondents.

The moral duty to purchase increased for all the considered groups. Women report significantly higher values than men for all the product categories (Figure 4b; Table A2). Significant differences are found among age groups for each product category (Figure 4c; Table A3). Respondents belonging to the group “25–34” increased their moral duty significantly less than respondents belonging to other groups for multiple types of products. On the contrary, the group “55–65” shows higher values than other groups. Results reveal significant differences among household income groups only for organic products (Figure 4d; Table A4). The “3.001–4.500” group shows a lower increase in the level of moral duty to purchase Organic products compared to the “<1.500” group. Concerning education, significant differences among groups are found on Made in Italy, Fairtrade, Organic, and Eco‐sustainable products: respondents with the lowest education level report a higher increase in their sense of moral duty to purchase these products (Figure 4e; Table A5).

### 4.5 Q5: Social influence to purchase sustainable products

Results show an increase of social influence for all product categories, as respondents indicated that people important to them think it is right to buy these products more than before the pandemic (Figure 5a). Local products, Made in Italy products, and Products sold by neighborhood stores report the highest increase with 47.23%, 42.41%, and 40.39% of the responses on the last two anchors of the scale (i.e, “Moderately” and “Extremely”), respectively.

All the considered groups report an increase. Women have significantly higher values than men for all the product categories (Figure 5b; Table A2). Concerning age groups, results reveal significant differences among the groups (Figure 5c; Table A3). For Organic and Fair Trade products the values of the group “25–34” are lower than those of groups “45–54”, “55–65”, and “>65.” Further, the group “25–34” presents the lowest values for five of the six product categories. Alternatively, group “>65” reports the highest values for five of the six product categories. No significant differences among the household income groups are found (see Table A4). Regarding education, results reveal significant differences among education groups for the last three products (i.e., D, E, and F) for which the “Middle school or lower” and “High school” groups have higher values than the other two groups (see Table A5).

### 4.6 Q6: Purchase frequency of sustainable products

Results show that more than 50% of respondents increased the frequency of purchase related to Made in Italy and Local products, around 50% of respondents increased the frequency of purchase of Products sold by neighborhood stores, while more than 20% of respondents increased the frequency of purchase of fair‐trade, organic, and eco‐sustainable products (Figure 6a). These results are consistent with those of the Coop Report (2020) and Severo et al. (2021), which showed how the pandemic has increased sustainable consumption, as well as with Castellini et al. (2021), which found that Italian consumers have frequently purchased certified sustainable food products during the pandemic period. Likewise, Sun et al. (2021) found that the emotional response induced by the Covid‐19 pandemic has driven Chinese consumers to increase their green consumption. Moreover, Ben Hassen et al. (2021) found that Bosnian consumers have increased the amounts of local food consumed.

The purchase frequency increased for all the socio‐demographic groups, as the mean values for each group are higher than three. Women increased their purchase frequency for all categories of products significantly more than men, similarly to the previous questions (Figure 6b; Table A2). This result is consistent with Li et al. (2021), who highlighted that Chinese women have increased the purchase of sustainable food more than men due to the Covid‐19 pandemic. Concerning the age groups, results do not reveal significant differences among groups (Figure 6c; Table A3). Alternatively, Li et al. (2021) found that Chinese people over 40 were more likely to purchase sustainable food than people under 40. Similarly, the study by Peluso et al. (2021) on Italian consumers highlighted that the older the consumers, the higher the willingness to increase environmentally sustainable purchases will be. Regarding the household income groups, significant differences are found only for organic products, where the “<1.500” group has a significantly higher value than the “3.001–4.500” group (Figure 6d; Table A4). This may be related to the fact that the “<1.500” income range has a significantly greater moral duty to purchase Organic products than the “3.001–4.500” income range, as well as more concern for environmental issues. With respect to education, no significant differences are reported (Figure 6e; Table A5).

### 4.7 Q7: Purchase behaviors

Results show several changes in purchase behaviors (Figure 7a). More than 50% of respondents increased the frequency of purchasing products online. This result is consistent with other recent studies conducted on several countries, such as Italy (Alaimo et al., 2020; Principato et al., 2020), Republic of Korea (Moon et al., 2021), the United States (Wang et al., 2020), China (Xiong et al., 2021), and Bosnia and Herzegovina (Ben Hassen et al., 2021). Around 50% of respondents avoided, more often, the purchase of non‐essential products, consistently with results by Mehta et al. (2020). This behavior is typical of the phases after a destructive event (Forbes, 2017). Further, around 50% of respondents limited the number of times they shop in physical stores buying large quantities at a time. This result is consistent with other studies conducted on Italian (Vittuari et al., 2021), United States (Wang et al., 2020), and German (Schmidt et al., 2021) consumers. Around 25% of respondents decreased the frequency of shopping in large stores, a result consistent with Moon et al. (2020) and Rodgers et al. (2021). Finally, around 30% of respondents increased the frequency of reading product labels.

Concerning the results related to the specific groups, it can be noted that women reported significantly higher values than men for all the purchase behaviors, except for Purchasing products online and Shopping in large stores (Figure 7b; Table A2). Regarding item D, our results are consistent with Vittuari et al. (2021), who found that, compared to men, women buy more groceries at once for their household per shopping trip and reduce the shopping frequency more. Results reveal significant differences among the age groups on items B and C (Figure 7c; Table A3). Respondents under 45 (groups “18–24”, “25–34”, and “35–44”) have increased the frequency of shopping online significantly more than older respondents. This result might be due to the higher familiarity of young people with online platforms. Respondents over 35 (groups “35–44”, “45–54”, “55–65”) have reduced the frequency of shopping in large stores significantly more than the younger respondents, probably because they are more vulnerable to Covid‐19 and shopping in large stores is perceived as a situation characterized by potential infection risks. Regarding the household income, no significant differences can be found among groups, except for purchasing products online (Figure 7d; Table A4). Respondents with a net monthly household income higher than 3.000 euros increased the frequency of online shopping significantly more than other respondents whose net income is lower than 3.000 euros. This result may be linked to the greater economic availability and ease of access to e‐commerce for high‐income people. Relating to education (see Table A5), respondents belonging to the “Middle school or lower” group increased the frequency of online shopping significantly less than the other respondents, perhaps due to a lower level of familiarity with online platforms. The “High school” group increased the frequency of checking the ingredients or materials used on the product labels significantly more than the “Postgraduate course or PhD” group.

### 4.8 Q8: Pro‐environmental behaviors

Over 20% of respondents increased the frequency of carrying out pro‐environmental behaviors due to the Covid‐19 pandemic (Figure 8a). This could be linked to the increased awareness of the impact of human behavior on the environment, as well as to the concern for environmental problems. Concerning items B and C, several authors found similar results, reporting a decrease in food wastage during the Covid‐19 pandemic by Italian (Borsellino et al., 2020; Pappalardo et al., 2020; Principato et al., 2020; Rodgers et al., 2021), United States (Rodgers et al., 2021), Tunisian (Jribi et al., 2020), Romanian (Burlea‐Schiopoiu et al., 2021), Bosnian (Ben Hassen et al., 2021), and Brazilian and Portuguese (Severo et al., 2020) consumers. Further, findings are consistent with Mehta et al. (2020), who highlighted how families are more careful about recycling and the environment due to the Covid‐19 pandemic.

The frequency of carrying out pro‐environmental behaviors increased for all groups, as the mean values for each group are higher than three. It can be noted that women have increased, statistically more than men, the frequency of adopting zero‐waste behavior and avoiding buying packaged products (Figure 8b; Table A2). Similarly, Rodgers et al. (2021) found that women are more likely to reduce food waste compared to men. This result could be related to the higher increase in the environmental concern and awareness shown by women. Results do not reveal significant differences among the age groups (Figure 8c; Table A3). Concerning household income (Figure 8d; Table A4), results show that respondents belonging to the “<1.500” group increased the frequency in avoiding waste energy at home significantly more than respondents belonging to the “>4.500” group. This may be related to the economic savings that people can achieve thanks to this practice. Further, the “1.500–3.000” group shows significantly higher values than the “>4.500” group concerning the frequency of doing the separate collection of waste. This may be due to the fact that, as shown in Figure 2d, respondents belonging to the “1.500–3.000” group significantly increased the concern related to the disposal of undifferentiated wastes. Regarding education, no significant differences are found among groups (see Table A5).

### 4.9 Q9: Use of transportation means

Results highlight several changes in the use of transportation means. Over 40% of the respondents decreased the frequency of public transport use. This result is consistent with Anke et al. (2021), König and Dreßler (2021), Xiong et al. (2021), and Degli Esposti et al. (2021). Similarly, around 22% of respondents reduced the frequency of using car‐sharing, a result consistent with the studies by Degli Esposti et al. (2021) and Wang et al. (2021). Both the above‐mentioned results could be related to the high risk of contagion associated with these means of transport. The overall use of cars seems not changed, resulting not in line with the study of Anke et al. (2021), Xiong et al. (2021), and Wang et al. (2021), which found an increase in the car use by German, Chinese, and United States customers due to the pandemic. Around 20% of respondents increased the frequency of using sustainable means of transport (e.g., bicycle). Such a behavior could have been affected by the economic incentives provided by the Italian government,^5^ aimed at increasing the use of these means of transport. Similarly, Wang et al. (2021) and Xiong et al. (2021) found that consumers in the United States and China have increased the use of bikes due to the Covid‐19 pandemic. Finally, 48% of the respondents indicated an increase in the frequency of walking. This result is consistent with Abdullah et al. (2020), Anke et al. (2021), Xiong et al. (2021), and Wang et al. (2021), whose analyses involved various countries of the world. The increase in walking and in the use of sustainable transportation means may be due to the fact that these are safer alternatives, in terms of contamination risk, to public transport.

Concerning the results related to the specific groups, significant differences in *gender* are reported only in the last statement (Figure 9b; Table A2): women increased the frequency of walking significantly more than men. Results show significant differences among the age groups in the Use of public transport and in the Use of car (Figure 9c; Table A3). The youngest group (“18–24”) reduced the frequency of using public transport significantly more than the “45–54” group. Differently from the other groups, respondents with ages between 18 and 34 years (“18–24” and “25–34” groups) increased the frequency of car use. In this regard, the (partial) closure of schools and universities (which have mostly adopted distance learning methods) could have contributed to reducing the use of public transport by young people. Furthermore, young people could have moved from public transport to car usage. Similarly, Xiong et al. (2021) found that young people have reduced the use of public transport more compared to older people, probably due to the higher use of this means of transport by younger consumers before the pandemic. Regarding the household income, respondents with a net monthly income higher than 3.000 euros increased the frequency of using sustainable means of transport more compared to respondents with a net monthly income lower than 1.500 euros, who have slightly reduced such a frequency (Figure 9d; Table A4). This result might be due to the economic availability to buy these means of transport. In contrast, Xiong et al. (2021) found that the higher the income, the smaller the increase in the use of bicycle and e‐bike. Respondents belonging to the group “3.001–4.500” have decreased the frequency of car‐sharing less than respondents belonging to the “<1.500” group. Concerning education (see Table A5), results revealed significant differences among groups only for the frequency of car‐sharing, for which the group “Elementary or Middle school” displays a lower value.

### 4.10 Q10: Willingness to pay a premium price for sustainable products

Results show that, depending on the specific category of sustainable product, between 20% and 45% of the respondents indicated an increase in the willingness to pay a premium price due to the pandemic (Figure 10a). Alternatively, between 3% and 6% of respondents decreased their willingness to pay. Between 51% and 71% of respondents did not change their willingness to pay.

The willingness to pay a premium price increased for all socio‐demographic groups, as the mean values for each group are higher than three. Significant differences between men and women are found only for two product categories, that is, made in Italy and organic products, for which women reported a significantly higher increase in the willingness to pay a premium price (Figure 10b; Table A2). This could be related to the fact that women increased the frequency to read product labels more than men. This result is consistent with Shahsavar and Kube (2020), who found that women are willing to pay more for eco‐friendly products. No significant differences among the age groups are found (Figure 10c; Table A3). With respect to household income (see Table A4), no significant differences among the groups are found. However, observing Figure 10d, the highest income range displays the highest values for all the categories of products. This is in line with what was found by Witek and Kuźniar (2021): the higher the economic availability, the higher the consumer intention to purchase green products. Relating to *education*, no significant differences among groups are found (see Table A5).

## 5 IMPLICATIONS

Based on the obtained results, several implications can be developed at multiple levels: theoretical (Section 5.1), managerial (Section 5.2), and policy‐related (Section 5.3).

### 5.1 Theoretical implications

In terms of theoretical implications, this study contributes to enhanced scholarly knowledge at the intersection between the consumer dynamics literature (Baumert et al., 2020; Forbes, 2017; Kirk & Rifkin, 2020; Sneath et al., 2009; Zwanka & Buff, 2021) and the green/sustainable consumer behavior literature (e.g., Alzubaidi et al., 2020; Dangelico et al., 2021; Diamantopoulos et al., 2003; Luchs & Mooradian, 2012; Sheoran & Kumar, 2021; Stagl & O'Hara, 2002), by assessing the influence of an unexpected and disruptive event ‐ the Covid‐19 pandemic ‐ on sustainable consumer behavior, and comparing the behavior of different socio‐demographic groups. Limited research has been conducted on this topic so far (e.g., Borsellino et al., 2020; Jian et al., 2020; Qi et al., 2020; Severo et al., 2020). Indeed, the literature called to further investigate whether the Covid‐19 pandemic is inspiring pro‐environmental attitudes and to better understand the impact of the pandemic on environmental concerns and sustainable consumer behavior (e.g., Jian et al., 2020; Peluso et al., 2021; Sarkis et al., 2020).

Accordingly, this study analyzes the effect of the Covid‐19 pandemic on sustainable consumer behavior, considering several aspects (i.e., psychological factors, consumption patterns, lifestyle habits), thus providing a comprehensive view of the phenomenon under multiple perspectives. Specifically, this study explicitly focuses on the changes, generated by the pandemic, in sustainable consumer behavior (willingness to pay more for sustainable products, frequency of purchase of sustainable products, purchase behavior and habits) as well as in its determinants (awareness, concern, moral duty, social influence) simultaneously. Results highlight that the Covid‐19 pandemic actually drove relevant changes in sustainable consumer behavior, leading to an increase in sustainable behavior, and that these changes are affected by consumers' socio‐demographic characteristics. Moreover, this study considers in the purchase behavior the economic, environmental, and social dimensions of sustainability simultaneously, by including different product categories (i.e., Sold by neighborhood stores, Made in Italy, Local, Fairtrade, Organic, and Eco‐sustainable products) related to the three above‐mentioned aspects of sustainability. This is a novelty compared to previous studies. Our results show that the increase in the sense of moral duty to buy, the social pressure to buy, and the purchase frequency are way higher for specific categories of sustainable products (i.e., Sold by neighborhood stores, Made in Italy, Local). This implies that, when studying sustainable consumer behavior and/or the effect of catastrophic events on it, rather than generally referring to sustainable consumer behavior or to sustainable products, it would be more appropriate to be specific on the category/categories of products under investigation.

Furthermore, this study offers elements of novelty, also with regard to the context in which it has been carried out. Actually, previous research conducted on the Italian context regarding the impact of the Covid‐19 pandemic on consumer behavior is quite limited and focused on very specific aspects, such as sustainable consumption (Castellini et al., 2021; Di Renzo et al., 2020; Peluso et al., 2021), food habits—including food waste (Amicarelli et al., 2021; Cai et al., 2021; Principato et al., 2020)—and shopping patterns (Degli Esposti et al., 2021; Vittuari et al., 2021). Alternatively, our study considers multiple aspects of sustainable consumer behavior, providing a broader picture of the phenomenon. Furthermore, our research also analyzes and compares the changes in consumer behavior for different socio‐demographic groups, highlighting that the way in which Italian consumers changed their behavior due to the Covid‐19 pandemic is strongly affected by their socio‐demographic characteristics.

### 5.2 Managerial implications

In terms of managerial implications, our study provides useful insights for companies. Specifically, our results show a general increase in both the frequency of purchase and the willingness to pay more for several categories of sustainable products. These changes in consumer behavior highlight growing opportunities for companies to embrace sustainability into their strategies, position themselves as sustainable firms, and develop sustainable products. Further, the increase in the frequency of purchase and willingness to pay more for made in Italy and local products (higher than that of the other considered product categories), as well as the higher consumers' attention to reading product labels to check the place of production, may let companies change their production strategies (if production processes are currently delocalized) or better promote these characteristics (if products are already manufactured locally or in Italy).

Local production and short supply chain strategies could be beneficial for companies also from a supply risk management perspective. Indeed, the Covid‐19 pandemic has strongly impacted global industries causing supply chain disruptions and uncertainties (Cankurtaran & Beverland, 2020; Kumar & Sharma, 2021). As a result, companies have had to cope with a lack of supplies, caused by the contraction of international trade, the closure of supplier companies due to the economic crisis, and the restrictions posed by governments. Thus, companies should leverage on the preferences of consumers for local products, fostered by the Covid‐19 pandemic, to reorganize their supply chains. In particular, short supply chains would allow companies to achieve a two‐fold objective even after the end of the pandemic: meet the growing consumers' demand for local products and reduce supply chain management risks. This would in turn lead to a reduction of the supply chain environmental impact compared to that caused by global supply chains.

Moreover, the higher consumers' attention to reading product labels to check ingredients or materials used should encourage companies to carefully choose them, using high‐quality materials and ingredients. The reduction of consumers' purchase of packaged products, to avoid plastic consumption, should stimulate firms to invest in alternative packaging design or innovative ways of selling products (i.e., draft products).

Furthermore, this study results give in‐depth insights on the purchasing behavior of consumers under various aspects, which can be useful in developing marketing strategies. For instance, given the large increase in online purchases, companies should strengthen their online presence by investing in the implementation/improvement of online sales channels. At the same time, the significant drop of purchases in physical stores might suggest, to companies that prefer selling through this channel, to reduce the (potential) negative perception associated with in‐store purchases and to design additional and exclusive benefits for customers buying in‐store, so as to re‐attract customers.

Further, the differences that emerged among the socio‐demographic groups highlight that specific marketing mixes should be developed for each category. For instance, the higher increase for women (compared to men) of the willingness to pay more for organic products may suggest companies to develop high‐quality organic product lines and advertising campaigns specifically addressed to this segment. Similarly, the higher increase for women of the willingness to pay a premium price for made in Italy products may encourage companies to develop high‐quality made in Italy product lines and give more emphasis on the place of production in advertisements addressed to this category of consumers. The higher increase in online purchasing by younger consumers may suggest companies to leverage even more on this sales channel when developing marketing mixes for young people.

In sum, this study highlights that changes induced by the Covid‐19 pandemic may represent an opportunity for companies to redefine their production and marketing strategies in a sustainable way, to meet the needs of consumers who are growingly concerned about sustainability issues.

### 5.3 Policy‐related implications

Several implications for policymakers can be derived from this study's results. First, this study offers an overview of the changes in consumers' environmental awareness and concern due to Covid‐19, highlighting differences among socio‐demographic groups. For instance, respondents between 25 and 44 y.o. reported the lowest increase in the concern for environmental problems and in the awareness of the environmental impact of their purchase choices and behavior. Similar results were found for the higher income range group, which reported the lowest values in the increase of the frequency of recycling and reduction of energy waste. These results, if associated with low initial absolute values, can be useful to develop information campaigns aimed at raising environmental awareness of specific groups. Second, the results showed that the lower‐income range has increased the use of sustainable means of transport (e.g., bicycle) less than the other income groups, despite the economic incentives issued by the Italian government. Policymakers should consider this result when revising the structure of incentives, aimed at increasing the efficacy even for low‐income consumers. Finally, given the significant decrease in the use of public transport, it would be advisable to improve the safety of public means of transport, for example by increasing the number of urban busses per route, so reducing the used capacity per bus, ceteris paribus.

## 6 LIMITATIONS, FUTURE RESEARCH DIRECTIONS, AND CONCLUSION

This article studied the extent of changes in sustainable consumer behavior caused by the Covid‐19 pandemic in Italy. Results demonstrated that the Covid‐19 pandemic has indeed caused several changes in sustainable consumer behavior and that these changes are different according to socio‐demographic characteristics of respondents. However, this research has some limitations.

First, this study uses a convenience sample and most of the respondents are young Italian people ‐ around 50% of respondents are under 35. Thus, caution should be made in generalizing to the whole Italian population. Future studies could involve a sample with a better representation of all age groups, as well as be conducted in multiple geographical areas, so as to allow for cross‐country comparisons.

Second, social desirability bias could have affected questionnaire answers, since some questions referred to socially desirable behaviors, like sustainable behaviors (Fisher, 1993; Luchs et al., 2010; Steenkamp et al., 2010). Further, our results showed that women have significantly higher values than men for most of the investigated aspects. These results could be related to the fact that women are more prone to report socially desirable behaviors than men (Dalton & Ortegren, 2011) or to a higher attitude of women towards environmental issues (Davidson & Freudenburg, 1996; Diamantopoulos et al., 2003; Luchs & Mooradian, 2012). In order to reduce the risk of social desirability bias, we clearly stated to participants, at the beginning of the questionnaire, that data would have been collected anonymously, as well as that there were no correct or wrong answers; further, no data that could have been used to identify respondents (e.g., name, e‐mail) were collected. However, future studies could be devoted to more deeply investigating the underlying motivations of gender‐related significant differences in sustainable consumer behavior changes due to the Covid‐19 pandemic.

Another limitation of this study is that we directly measured the extent of changes in sustainable consumer behavior without having knowledge about the baseline level (i.e., the behavior before the Covid‐19 pandemic). Further, this study is cross‐sectional. It would be interesting to repeat it at other points in time during the pandemic to assess the evolution of sustainable consumer behavior over time, as well as at the end of the pandemic, to understand whether the changes induced by the Covid‐19 pandemic are temporary or permanent.

In conclusion, this research offers a general picture of the changes in sustainable consumer behavior that occurred in the Italian context due to the Covid‐19 pandemic. Results have shown that this catastrophic and unexpected event led consumers to be more concerned about environmental problems, more aware of individual impacts, and to behave more sustainably.

## Supporting information


**Appendix** **S1**: Appendix tables.

## Endnotes

^1^
For eco‐sustainable products, local products, and fair‐trade products a brief description was provided.

^2^
Products sold in neighborhood stores have not been included in this question since, differently from the other considered categories, their sustainability only derives from the place where they are sold rather than from clearly recognizable intrinsic product characteristics.

^3^
It should be noticed that questions in the first and the second sections use different Likert‐type scales; the scale for Q1‐Q5 can only measure either no change or an increase, while the scale for Q6‐Q10 can measure a change in both directions (i.e., a decrease or an increase). As a result, mean values presented in Figures 1, 2, 3, 4, 5 and 6, 7, 8, 9, 10 have different meanings. In Figures 6, 7, 8, 9, 10, a dotted line represents the reference value for no change.

^4^
The questionnaire also included some other questions, out of the scope of this article.

^5^
The Italian government established the Mobility Bonus on May 4, 2020, valid until February15, 2021. It is a contribution for a maximum amount of 500 euros which covers up to 60% of the costs incurred to buy a sustainable means of transport (source: www.mit.gov.it).

## References

[sd2322-bib-0001] Abdullah, M. , Dias, C. , Muley, D. , & Shahin, M. (2020). Exploring the impacts of COVID‐19 on travel behavior and mode preferences. Transportation Research Interdisciplinary Perspectives, 8(12), 100255. 34173481 10.1016/j.trip.2020.100255 PMC7640923

[sd2322-bib-0002] Agadayi, E. , Nemmezi Karaca, S. , Ersen, G. , Ayhan Baser, D. , Küçükceran, H. , Bilgili, P. , & Küçük, İ. G. (2021). Breastfeeding frequency of primary healthcare professionals and effective factors. International Journal of Clinical Practice, 75, e14499. 34117668 10.1111/ijcp.14499

[sd2322-bib-0003] Alaimo, L. S. , Fiore, M. , & Galati, A. (2020). How the COVID‐19 pandemic is changing online food shopping human behaviour in Italy. Sustainability (Switzerland), 12(22), 9594.

[sd2322-bib-0004] Alzubaidi, H. , Slade, E. L. , & Dwivedi, Y. K. (2020). Examining antecedents of consumers' pro‐environmental behaviours: TPB extended with materialism and innovativeness. Journal of Business Research, 122, 685–699.

[sd2322-bib-0005] Amicarelli, V. , Lagioia, G. , Sampietro, S. , & Bux, C. (2021). Has the COVID‐19 pandemic changed food waste perception and behavior? Evidence from Italian consumers. Socio‐Economic Planning Sciences, in press, 101095. 10.1016/j.seps.2021.101095 PMC9751389 36536871

[sd2322-bib-0006] Anke, J. , Francke, A. , Schaefer, L. M. , & Petzoldt, T. (2021). Impact of SARS‐CoV‐2 on the mobility behaviour in Germany. European Transport Research Review, 13(1), 10. 10.1186/s12544-021-00469-3 PMC7835317 38624595

[sd2322-bib-0007] Barbarossa, C. , & De Pelsmacker, P. (2016). Positive and negative antecedents of purchasing eco‐friendly products: A comparison between green and non‐green consumers. Journal of Business Ethics, 134(2), 229–247.

[sd2322-bib-0008] Baumert, T. , de Obesso, M. M. , & Valbuena, E. (2020). How does the terrorist experience alter consumer behaviour? An analysis of the Spanish case. Journal of Business Research, 115, 357–364.

[sd2322-bib-0009] BCG (2020). The pandemic is heightening environmental awareness. https://www.bcg.com/publications/2020/pandemic-is-heightening-environmental-awareness

[sd2322-bib-0010] Beldad, A. , & Hegner, S. (2018). Determinants of fair trade product purchase intention of Dutch consumers according to the extended theory of planned behaviour. Journal of Consumer Policy, 41(3), 191–210.

[sd2322-bib-0011] Ben Hassen, T. , El Bilali, H. , Allahyari, M. S. , Karabašević, D. , Radosavac, A. , Berjan, S. , & Obhođaš, I. (2021). Food behavior changes during the COVID‐19 pandemic: Statistical analysis of consumer survey data from Bosnia and Herzegovina. Sustainability, 13(15), 8617.

[sd2322-bib-0012] Borsellino, V. , Kaliji, S. A. , & Schimmenti, E. (2020). COVID‐19 drives consumer behaviour and agro‐food markets towards healthier and more sustainable patterns. Sustainability, 12(20), 8366.

[sd2322-bib-0013] Brewer, P. , & Sebby, A. G. (2021). The effect of online restaurant menus on consumers' purchase intentions during the COVID‐19 pandemic. International Journal of Hospitality Management, 94, 102777. 34785837 10.1016/j.ijhm.2020.102777 PMC8588438

[sd2322-bib-0014] Burlea‐Schiopoiu, A. , Ogarca, R. F. , Barbu, C. M. , Craciun, L. , Baloi, I. C. , & Mihai, L. S. (2021). The impact of COVID‐19 pandemic on food waste behaviour of young people. Journal of Cleaner Production, 294, 126333. 34720458 10.1016/j.jclepro.2021.126333 PMC8541752

[sd2322-bib-0015] Cai, M. , Guy, C. , Héroux, M. , Lichtfouse, E. , & An, C. (2021). The impact of successive COVID‐19 lockdowns on people mobility, lockdown efficiency, and municipal solid waste. Environmental Chemistry Letters, 19, 3959–3965. 34366754 10.1007/s10311-021-01290-z PMC8325046

[sd2322-bib-0016] Cankurtaran, P. , & Beverland, M. B. (2020). Using design thinking to respond to crises: B2B lessons from the 2020 COVID‐19 pandemic. Industrial Marketing Management, 88, 255–260.

[sd2322-bib-0017] Castellini, G. , Savarese, M. , & Graffigna, G. (2021). The impact of COVID‐19 outbreak in Italy on the sustainable food consumption intention from a “one health” perspective. Frontiers in Nutrition, 8, 622122. 33791331 10.3389/fnut.2021.622122 PMC8006295

[sd2322-bib-0018] Coop Report (2020). Survey post Covid‐19—The new normality of Italians, 2020. https://www.italiani.coop/postcovid19-la-nuova-normalita-degli-italiani/.

[sd2322-bib-0019] Coulthard, H. , Sharps, M. , Cunliffe, L. , & van den Tol, A. (2021). Eating in the lockdown during the Covid 19 pandemic; self‐reported changes in eating behaviour, and associations with BMI, eating style, coping and health anxiety. Appetite, 161(1), 105082. 33476651 10.1016/j.appet.2020.105082 PMC7976455

[sd2322-bib-0020] Dalton, D. , & Ortegren, M. (2011). Gender differences in ethics research: The importance of controlling for the social desirability response bias. Journal of Business Ethics, 103(1), 73–93.

[sd2322-bib-0021] Dangelico, R. M. , Nonino, F. , & Pompei, A. . (2021). Which are the determinants of green purchase behaviour? A study of Italian consumers. Business Strategy and the Environment, 30(5), 2600–2620.

[sd2322-bib-0022] Davidson, D. J. , & Freudenburg, W. R. (1996). Gender and environmental risk concerns: A review and analysis of available research. Environment and Behavior, 28(3), 302–339.

[sd2322-bib-0023] Degli Esposti, P. , Mortara, A. , & Roberti, G. (2021). Sharing and sustainable consumption in the era of COVID‐19. Sustainability, 13(4), 1903.

[sd2322-bib-0024] Dhir, A. , Sadiq, M. , Talwar, S. , Sakashita, M. , & Kaur, P. (2021). Why do retail consumers buy green apparel? A knowledge‐attitude‐behaviour‐context perspective. Journal of Retailing and Consumer Services, 59, 102398.

[sd2322-bib-0025] Di Crosta, A. , Ceccato, I. , Marchetti, D. , La Malva, P. , Maiella, R. , Cannito, L. , & Di Domenico, A. (2021). Psychological factors and consumer behavior during the COVID‐19 pandemic. PLoS One, 16(8), e0256095. 34398916 10.1371/journal.pone.0256095 PMC8366984

[sd2322-bib-0026] Di Renzo, L. , Gualtieri, P. , Pivari, F. , Soldati, L. , Attinà, A. , Cinelli, G. , Cinelli, G. , Leggeri, C. , Caparello, G. , Barrea, L. , Scerbo, F. , Esposito, E. , & de Lorenzo, A. (2020). Eating habits and lifestyle changes during COVID‐19 lockdown: An Italian survey. Journal of Translational Medicine, 18(1), 1–15. 32513197 10.1186/s12967-020-02399-5 PMC7278251

[sd2322-bib-0027] Diamantopoulos, A. , Schlegelmilch, B. B. , Sinkovics, R. R. , & Bohlen, G. M. (2003). Can socio‐demographics still play a role in profiling green consumers? A review of the evidence and an empirical investigation. Journal of Business Research, 56(6), 465–480.

[sd2322-bib-0028] Fisher, R. J. (1993). Social desirability bias and the validity of indirect questioning. Journal of Consumer Research, 20(2), 303–315.

[sd2322-bib-0029] Forbes, S. L. (2017). Post‐disaster consumption: Analysis from the 2011 Christchurch earthquake. The International Review of Retail, Distribution and Consumer Research, 27(1), 28–42.

[sd2322-bib-0030] Gilal, F. G. , Chandani, K. , Gilal, R. G. , Gilal, N. G. , Gilal, W. G. , & Channa, N. A. (2020). Towards a new model for green consumer behaviour: A self‐determination theory perspective. Sustainable Development, 28(4), 711–722.

[sd2322-bib-0031] Herzenstein, M. , Horsky, S. , & Posavac, S. S. (2015). Living with terrorism or withdrawing in terror: Perceived control and consumer avoidance. Journal of Consumer Behaviour, 14(4), 228–236.

[sd2322-bib-0032] Jian, Y. , Yu, I. Y. , Yang, M. X. , & Zeng, K. J. (2020). The impacts of fear and uncertainty of covid‐19 on environmental concerns, brand trust, and behavioral intentions toward green hotels. Sustainability, 12(20), 8688.

[sd2322-bib-0033] Jribi, S. , Ismail, H. B. , Doggui, D. , & Debbabi, H. (2020). COVID‐19 virus outbreak lockdown: What impacts on household food wastage? Environment, Development and Sustainability, 22(5), 3939–3955. 32837271 10.1007/s10668-020-00740-y PMC7166255

[sd2322-bib-0034] Kang, J. , Liu, C. , & Kim, S. H. (2013). Environmentally sustainable textile and apparel consumption: The role of consumer knowledge, perceived consumer effectiveness and perceived personal relevance. International Journal of Consumer Studies, 37(4), 442–452.

[sd2322-bib-0035] Khan, M. S. , Saengon, P. , Alganad, A. M. N. , Chongcharoen, D. , & Farrukh, M. (2020). Consumer green behaviour: An approach towards environmental sustainability. Sustainable Development, 28(5), 1168–1180.

[sd2322-bib-0036] Kirk, C. P. , & Rifkin, L. S. (2020). I'll trade you diamonds for toilet paper: Consumer reacting, coping and adapting behaviors in the COVID‐19 pandemic. Journal of Business Research, 117, 124–131. 32834208 10.1016/j.jbusres.2020.05.028 PMC7241317

[sd2322-bib-0037] König, A. , & Dreßler, A. (2021). A mixed‐methods analysis of mobility behavior changes in the COVID‐19 era in a rural case study. European Transport Research Review, 13(1), 15. 10.1186/s12544-021-00472-8 PMC7873667 38624561

[sd2322-bib-0038] Kooli, K. , Tzempelikos, N. , Foroudi, P. , & Mazahreh, S. (2019). What drives B‐to‐B marketers in emerging countries to use social media sites? Journal of Business‐to‐Business Marketing, 26(3–4), 245–264.

[sd2322-bib-0039] Kumar, B. , & Sharma, A. (2021). Managing the supply chain during disruptions: Developing a framework for decision‐making. Industrial Marketing Management, 97, 159–172.

[sd2322-bib-0040] Li, S. , Kallas, Z. , & Rahmani, D. (2021). Did the COVID‐19 lockdown affect consumers' sustainable behaviour in food purchasing and consumption in China? Food Control, 132, 108352. 10.1016/j.foodcont.2021.108352 PMC9716012 36474958

[sd2322-bib-0041] Luchs, M. G. , & Mooradian, T. A. (2012). Sex, personality, and sustainable consumer behaviour: Elucidating the gender effect. Journal of Consumer Policy, 35(1), 127–144.

[sd2322-bib-0042] Luchs, M. G. , Naylor, R. W. , Irwin, J. R. , & Raghunathan, R. (2010). The sustainability liability: Potential negative effects of ethicality on product preference. Journal of Marketing, 74(5), 18–31.

[sd2322-bib-0043] Magnier, L. , Mugge, R. , & Schoormans, J. (2019). Turning ocean garbage into products–Consumers' evaluations of products made of recycled ocean plastic. Journal of Cleaner Production, 215, 84–98.

[sd2322-bib-0044] Mainardes, E. W. , Espanhol, C. A. , & Cruz, P. B. D. (2021). Green consumption: Consumer behavior after an environmental tragedy. Journal of Environmental Planning and Management, 64(7), 1156–1183.

[sd2322-bib-0045] Martínez‐de‐Quel, Ó. , Suárez‐Iglesias, D. , López‐Flores, M. , & Pérez, C. A. (2021). Physical activity, dietary habits and sleep quality before and during COVID‐19 lockdown: A longitudinal study. Appetite, 158(7), 105019. 33161046 10.1016/j.appet.2020.105019 PMC8580211

[sd2322-bib-0046] Mehta, S. , Saxena, T. , & Purohit, N. (2020). The new consumer behaviour paradigm amid COVID‐19: Permanent or transient? Journal of Health Management, 22(2), 291–301.

[sd2322-bib-0047] Miao, L. , Im, J. , Fu, X. , Kim, H. , & Zhang, Y. E. (2021). Proximal and distal post‐COVID travel behavior. Annals of Tourism Research, 88, 103159.

[sd2322-bib-0048] Moon, J. , Choe, Y. , & Song, H. (2021). Determinants of consumers' online/offline shopping behaviours during the COVID‐19 pandemic. International Journal of Environmental Research and Public Health, 18(4), 1593. 33567566 10.3390/ijerph18041593 PMC7914819

[sd2322-bib-0049] Moser, A. K. (2015). Thinking green, buying green? Drivers of pro ‐ environmental purchasing behavior. Journal of Consumer Marketing, 32(3), 167–175.

[sd2322-bib-0050] Murphy, B. , Benson, T. , McCloat, A. , Mooney, E. , Elliott, C. , Dean, M. , & Lavelle, F. (2021). Changes in consumers' food practices during the covid‐19 lockdown, implications for diet quality and the food system: A cross‐continental comparison. Nutrients, 13(1), 20. 10.3390/nu13010020 PMC7822477 33374619

[sd2322-bib-0051] Noy, C. (2008). Sampling knowledge: The hermeneutics of snowball sampling in qualitative research. International Journal of Social Research Methodology, 11(4), 327–344.

[sd2322-bib-0052] Pappalardo, G. , Cerroni, S. , Nayga, R. M., Jr. , & Yang, W. (2020). Impact of Covid‐19 on household food waste: The case of Italy. Frontiers in Nutrition, 7, 585090. 33344492 10.3389/fnut.2020.585090 PMC7738320

[sd2322-bib-0053] Peluso, A. M. , Pichierri, M. , & Pino, G. (2021). Age‐related effects on environmentally sustainable purchases at the time of COVID‐19: Evidence from Italy. Journal of Retailing and Consumer Services, 60, 102443.

[sd2322-bib-0054] Principato, L. , Secondi, L. , Cicatiello, C. , & Mattia, G. (2020). Caring more about food: The unexpected positive effect of the Covid‐19 lockdown on household food management and waste. Socio‐Economic Planning Sciences, in press. 10.1016/j.seps.2020.100953 PMC9192149 35721383

[sd2322-bib-0055] Qi, X. , Yu, H. , & Ploeger, A. (2020). Exploring influential factors including COVID‐19 on green food purchase intentions and the intention–behaviour gap: A qualitative study among consumers in a Chinese context. International Journal of Environmental Research and Public Health, 17(19), 7106. 10.3390/ijerph17197106 PMC7579444 32998292

[sd2322-bib-0056] Rasool, S. , Rehman, A. , Cerchione, R. , & Centobelli, P. (2021). Evaluating consumer environmental behavior for sustainable development: A confirmatory factor analysis. Sustainable Development, 29(2), 318–326.

[sd2322-bib-0057] Rodgers, R. F. , Lombardo, C. , Cerolini, S. , Franko, D. L. , Omori, M. , Linardon, J. , & Fuller‐Tyszkiewicz, M. (2021). “Waste not and stay at home” evidence of decreased food waste during the COVID‐19 pandemic from the US and Italy. Appetite, 160, 105110. 33428972 10.1016/j.appet.2021.105110 PMC9755822

[sd2322-bib-0058] Rousseau, S. , & Deschacht, N. (2020). Public awareness of nature and the environment during the COVID‐19 crisis. Environmental and Resource Economics, 76(4), 1149–1159. 32836836 10.1007/s10640-020-00445-w PMC7354367

[sd2322-bib-0059] Safara, F. (2020). A computational model to predict consumer behaviour during COVID‐19 pandemic. Computational Economics. 10.1007/s10614-020-10069-3 PMC7643087 33169049

[sd2322-bib-0060] Sarkis, J. , Cohen, M. J. , Dewick, P. , & Schröder, P. (2020). A brave new world: Lessons from the COVID‐19 pandemic for transitioning to sustainable supply and production. Resources, Conservation and Recycling, 159(4), 104894. 32313383 10.1016/j.resconrec.2020.104894 PMC7164912

[sd2322-bib-0061] Schmidt, S. , Benke, C. , & Pané‐Farré, C. A. (2021). Purchasing under threat: Changes in shopping patterns during the COVID‐19 pandemic. PLoS One, 16(6), e0253231. 34106996 10.1371/journal.pone.0253231 PMC8189441

[sd2322-bib-0062] Severo, E. A. , de Guimarães, J. C. F. , & Dellarmelin, M. L. (2021). Impact of the COVID‐19 pandemic on environmental awareness, sustainable consumption and social responsibility: Evidence from generations in Brazil and Portugal. Journal of Cleaner Production, 286, 124947. 33173257 10.1016/j.jclepro.2020.124947 PMC7644235

[sd2322-bib-0063] Shahsavar, T. , & Kube, V. (2020). Willingness to pay for eco‐friendly furniture based on demographic factors. Journal of Cleaner Production, 250, 119466.

[sd2322-bib-0064] Sheoran, M. , & Kumar, D. (2021). Conceptualisation of sustainable consumer behaviour: Converging the theory of planned behaviour and consumption cycle. Qualitative Research in Organizations and Management: An International Journal, 17(1), 103–135.

[sd2322-bib-0065] Sneath, J. Z. , Lacey, R. , & Kennett‐Hensel, P. A. (2009). Coping with a natural disaster: Losses, emotions, and impulsive and compulsive buying. Marketing Letters, 20(1), 45–60.

[sd2322-bib-0066] Stagl, S. , & O'Hara, S. U. (2002). Motivating factors and barriers to sustainable consumer behaviour. International Journal of Agricultural Resources, Governance and Ecology, 2(1), 75–88.

[sd2322-bib-0067] Steenkamp, J. B. E. , De Jong, M. G. , & Baumgartner, H. (2010). Socially desirable response tendencies in survey research. Journal of Marketing Research, 47(2), 199–214.

[sd2322-bib-0068] Sun, X. , Su, W. , Guo, X. , & Tian, Z. (2021). The impact of awe induced by COVID‐19 pandemic on green consumption behavior in China. International Journal of Environmental Research and Public Health, 18(2), 543. 10.3390/ijerph18020543 PMC7826881 33440719

[sd2322-bib-0069] Tamrin, S. I. , Norman, A. A. , & Hamid, S. (2021). Intention to share: the relationship between cybersecurity behaviour and sharing specific content in Facebook. Information Research: an international electronic journal, 26(1).

[sd2322-bib-0070] Vaishar, A. , & Šťastná, M. (2022). Impact of the COVID‐19 pandemic on rural tourism in Czechia preliminary considerations. Current Issues in Tourism, 25(2), 187–191.

[sd2322-bib-0071] Vittuari, M. , Masotti, M. , Iori, E. , Falasconi, L. , Toschi, T. G. , & Segrè, A. (2021). Does the COVID‐19 external shock matter on household food waste? The impact of social distancing measures during the lockdown. Resources, Conservation and Recycling, 174, 105815. 10.1016/j.resconrec.2021.105815 PMC9758647 36569117

[sd2322-bib-0072] Wang, D. , He, B. Y. , Gao, J. , Chow, J. Y. , Ozbay, K. , & Iyer, S. (2021). Impact of COVID‐19 behavioral inertia on reopening strategies for new York City transit. International Journal of Transportation Science and Technology, 10(2), 197–211.

[sd2322-bib-0073] Wang, Y. , Xu, R. , Schwartz, M. , Ghosh, D. , & Chen, X. (2020). COVID‐19 and retail grocery management: Insights from a broad‐based consumer survey. IEEE Engineering Management Review, 48(3), 202–211.

[sd2322-bib-0074] Witek, L. , & Kuźniar, W. (2021). Green purchase behavior: The effectiveness of sociodemographic variables for explaining green purchases in emerging market. Sustainability, 13(1), 209.

[sd2322-bib-0075] Xiong, J. , Tang, Z. , Zhu, Y. , Xu, K. , Yin, Y. , & Xi, Y. (2021). Change of consumption Behaviours in the pandemic of COVID‐19: Examining residents' consumption expenditure and driving determinants. International Journal of Environmental Research and Public Health, 18(17), 9209. 34501796 10.3390/ijerph18179209 PMC8431450

[sd2322-bib-0076] Yang, Z. , Choe, Y. , Martell, M. , Yang Z. , Choe Y. & Martell M. (2021). COVID‐19 economic policy effects on consumer spending and foot traffic in the U.S. Journal of Safety Science and Resilience, 2(4), 230–237. 10.1016/j.jnlssr.2021.09.003 PMC8491973 40477778

[sd2322-bib-0077] Young, W. , Hwang, K. , McDonald, S. , & Oates, C. J. (2010). Sustainable consumption: Green consumer behaviour when purchasing products. Sustainable Development, 18(1), 20–31.

[sd2322-bib-0078] Zhang, J. Z. , & Chang, C. W. (2021). Consumer dynamics: Theories, methods, and emerging directions. Journal of the Academy of Marketing Science, 49(1), 166–196.

[sd2322-bib-0079] Zwanka, R. J. , & Buff, C. (2021). COVID‐19 generation: A conceptual framework of the consumer behavioral shifts to be caused by the COVID‐19 pandemic. Journal of International Consumer Marketing, 33(1), 58–67.

