# Supplementary material for: Is Covid‐19 changing sustainable consumer behavior? A survey of Italian consumers
Source: Sustainable Development. 2022 Apr 27:10.1002/sd.2322. Online ahead of print. doi: 10.1002/sd.2322 (PMC9111117; doi:10.1002/sd.2322)
Supplement: Supplementary file 1 — Appendix S1: Appendix tables. [file SD-9999-0-s001.docx]

**APPENDIX**

**Table A1 - Questionnaire**

| **Question** | **Statement** | **Reference** |
| --- | --- | --- |
| Q1 | Please indicate how much the Covid-19 pandemic has made you more aware that: | Adapted from BCG (2020) |
|  | A.      Humans can be threatened by environmental degradation |  |
|  | B.       The climate is threatened by human activities |  |
|  | C.       The different countries of the world are deeply interconnected | New item |
|  | D.      Global problems can be tackled with cooperation between different countries | New item |
|  | E.       Collaboration between different social actors is important to cope with complex situations | New item |
|  | F.       Cooperation between different political groups is important to cope with complex situations | New item |
| Q2 | Please indicate how much, following the Covid-19 pandemic, your concern has increased in the following aspects: | Adapted from BCG (2020) |
|  | A.      Infectious diseases |  |
|  | B.       Pollution (air, soil, and water) |  |
|  | C.       Climate change |  |
|  | D.      Unsustainable management of natural resources |  |
|  | E.       Natural habitat destruction |  |
|  | F.       Biodiversity loss |  |
|  | G.      Disposal of undifferentiated waste |  |
| Q3 | Please indicate how much the Covid-19 pandemic has made you more aware of the following aspects: | Adapted from Kang et al. (2013) |
|  | A.      My purchasing choices can have a significant impact on the environment |  |
|  | B.       My purchasing choices can have a significant impact on society |  |
|  | C.       My purchasing choices can have a significant impact on the economy |  |
|  | D.      My behavior can make a significant difference in mitigating environmental problems |  |
|  | E.       My behavior can make a significant difference in mitigating social problems |  |
|  | F.       My behavior can make a significant difference in mitigating the economic problems of my territory |  |
|  | G.      My behavior can make a significant difference in mitigating the economic problems of Italy |  |
| Q4 | Please indicate how much, following the Covid-19 pandemic, your sense of moral duty to purchase the following products has increased: | Adapted from Beldad and Hegner (2018), with new product categories |
|  | A.      Products sold by neighbourhood stores |  |
|  | B.       Made in Italy products |  |
|  | C.       Local products |  |
|  | D.      Fair-trade products |  |
|  | E.       Organic products |  |
|  | F.       Eco-sustainable products |  |
| Q5 | Please indicate how much, following the Covid-19 pandemic, people important to you (e.g., relatives, partners, friends, colleagues, public figures) think it is right to buy more than before the following products: | Adapted from Alzubaidi et al. (2020), with new product categories |
|  | A.       Products sold by neighbourhood stores |  |
|  | B.       Made in Italy products |  |
|  | C.       Local products |  |
|  | D.      Fair-trade products |  |
|  | E.       Organic products |  |
|  | F.       Eco-sustainable products |  |
| Q6 | *Product Type* - Please indicate how much the frequency of your actions has changed as compared to before the Covid-19 pandemic: | Adapted from BCG (2020), with new product categories |
|  | A.     Purchase of products sold by neighbourhood stores |  |
|  | B.     Purchase of Made in Italy products |  |
|  | C.     Purchase of Local products |  |
|  | D.     Purchase of Fair-trade products |  |
|  | E.     Purchase of Organic products |  |
|  | F.     Purchase of Eco-sustainable products |  |
| Q7 | *Purchase Behaviors* - Please indicate how much the frequency of your actions has changed as compared to before the Covid-19 pandemic: | Adapted from BCG (2020) |
|  | A.   Avoiding the purchase of non-essential products | New item |
|  | B.    Purchasing products online | New item |
|  | C.    Shopping in large stores | New item |
|  | D.    Limiting the number of times you shop in physical stores buying large quantities at a time | New item |
|  | E.  Reading product labels about the ingredients or       materials used |  |
|  | F.       Reading product labels to check the place of production |  |
| Q8 | *Pro-Environmental behaviors* - Please indicate how much the frequency of your actions has changed as compared to before the Covid-19 pandemic: | Adapted from BCG (2020) |
|  | A.     Avoid energy waste at home |  |
|  | B.      Do the separate collection of waste |  |
|  | C.      Try to adopt zero waste behavior |  |
|  | D.      Avoid buying packaged products to reduce my plastic consumption |  |
| Q9 | *Use of transportation means* - Please indicate how much the frequency of your actions has changed as compared to before the Covid-19 pandemic: | Adapted from BCG (2020) |
|  | A.      Use of sustainable means of transportation (e.g., bicycle) |  |
|  | B.       Use of car-sharing | New item |
|  | C.       Use of public transport | New item |
|  | D.       Use of car | New item |
|  | E.       Walking | New item |
| Q10 | Please indicate how much, compared to before the Covid-19 pandemic, your willingness to pay a premium price for the following product types has changed: | Adapted from Moser (2015), with new product categories |
|  | A.      Made in Italy products |  |
|  | B.       Local product |  |
|  | C.       Fair-trade products |  |
|  | D.      Organic products |  |
|  | E.       Eco-sustainable products |  |

**Table A2 - Gender comparison. Results of the Kruskal-Wallis test.**

| **Question** | | **Means** | | | **Sign**  **Kruskal Wallis test statistic** | |
| --- | --- | --- | --- | --- | --- | --- |
|  | **Item** | *Man* |  | *Woman* | $\chi{2\left( 1 \right)}$ | *p* |
| Q1 | Q1-A | 3,12 | < | 3,54 | 34,445 | <,001 |
|  | Q1-B | 3,37 | < | 3,77 | 30,220 | <,001 |
|  | Q1-C | 3,38 | < | 3,61 | 11,475 | ,001 |
|  | Q1-D | 3,59 | < | 3,85 | 11,541 | ,001 |
|  | Q1-E | 3,57 | < | 3,86 | 19,481 | <,001 |
|  | Q1-F | 3,35 | < | 3,77 | 29,439 | <,001 |
| Q2 | Q2-A | 3,32 | < | 3,65 | 38,465 | <,001 |
|  | Q2-B | 2,67 | < | 3,19 | 70,351 | <,001 |
|  | Q2-C | 2,65 | < | 3,16 | 62,216 | <,001 |
|  | Q2-D | 2,64 | < | 3,08 | 47,992 | <,001 |
|  | Q2-E | 2,79 | < | 3,32 | 60,860 | <,001 |
|  | Q2-F | 2,51 | < | 3,03 | 61,766 | <,001 |
|  | Q2-G | 2,82 | < | 3,41 | 71,144 | <,001 |
| Q3 | Q3-A | 2,61 | < | 3,18 | 74,541 | <,001 |
|  | Q3-B | 2,78 | < | 3,16 | 37,952 | <,001 |
|  | Q3-C | 2,98 | < | 3,32 | 29,977 | <,001 |
|  | Q3-D | 2,75 | < | 3,33 | 73,603 | <,001 |
|  | Q3-E | 2,76 | < | 3,29 | 72,257 | <,001 |
|  | Q3-F | 2,82 | < | 3,27 | 56,147 | <,001 |
|  | Q3-G | 2,61 | < | 3,05 | 50,903 | <,001 |
| Q4 | Q4-A | 2,89 | < | 3,41 | 67,955 | <,001 |
|  | Q4-B | 2,95 | < | 3,54 | 81,991 | <,001 |
|  | Q4-C | 3,19 | < | 3,68 | 54,514 | <,001 |
|  | Q4-D | 2,44 | < | 2,90 | 55,186 | <,001 |
|  | Q4-E | 2,46 | < | 2,96 | 57,212 | <,001 |
|  | Q4-F | 2,54 | < | 3,04 | 61,147 | <,001 |
| Q5 | Q5-A | 2,92 | < | 3,32 | 43,860 | <,001 |
|  | Q5-B | 2,95 | < | 3,34 | 39,165 | <,001 |
|  | Q5-C | 3,06 | < | 3,44 | 39,264 | <,001 |
|  | Q5-D | 2,39 | < | 2,76 | 40,292 | <,001 |
|  | Q5-E | 2,46 | < | 2,80 | 34,114 | <,001 |
|  | Q5-F | 2,48 | < | 2,79 | 30,944 | <,001 |
| Q6 | Q6-A | 3,41 | < | 3,60 | 19,624 | <,001 |
|  | Q6-B | 3,49 | < | 3,70 | 31,105 | <,001 |
|  | Q6-C | 3,56 | < | 3,76 | 22,013 | <,001 |
|  | Q6-D | 3,15 | < | 3,26 | 16,278 | <,001 |
|  | Q6-E | 3,20 | < | 3,33 | 15,352 | <,001 |
|  | Q6-F | 3,22 | < | 3,38 | 22,203 | <,001 |
| Q7 | Q7-A | 3,38 | < | 3,54 | 13,187 | <,001 |
|  | Q7-B | 3,74 | > | 3,67 | 0,549 | ,459 |
|  | Q7-C | 2,87 | > | 2,81 | 2,933 | ,087 |
|  | Q7-D | 3,34 | < | 3,55 | 28,036 | <,001 |
|  | Q7-E | 3,25 | < | 3,37 | 11,055 | ,001 |
|  | Q7-F | 3,29 | < | 3,42 | 14,339 | <,001 |
| Q8 | Q8-A | 3,28 | = | 3,28 | 0,325 | ,569 |
|  | Q8-B | 3,26 | < | 3,36 | 2,445 | ,118 |
|  | Q8-C | 3,21 | < | 3,27 | 5,538 | ,019 |
|  | Q8-D | 3,22 | < | 3,33 | 11,274 | ,001 |
| Q9 | Q9-A | 3,10 | > | 3,07 | 0,383 | ,536 |
|  | Q9-B | 2,72 | > | 2,67 | 0,421 | ,516 |
|  | Q9-C | 2,37 | > | 2,35 | 0,200 | ,655 |
|  | Q9-D | 3,06 | > | 2,97 | 2,923 | ,087 |
|  | Q9-E | 3,50 | < | 3,63 | 7,792 | ,005 |
| Q10 | Q10-A | 3,32 | < | 3,47 | 16,577 | <,001 |
|  | Q10-B | 3,44 | < | 3,49 | 1,550 | ,213 |
|  | Q10-C | 3,15 | < | 3,20 | 2,104 | ,147 |
|  | Q10-D | 3,16 | < | 3,28 | 15,693 | <,001 |
|  | Q10-E | 3,17 | < | 3,22 | 3,391 | ,066 |

**Table A3 - Age comparison. Results of the Kruskal-Wallis test and Dunn-Bonferroni Post hoc test.**

| **Means** | | | | | | | | **Sign.** | | |
| --- | --- | --- | --- | --- | --- | --- | --- | --- | --- | --- |
| **Question** | | **Group1** | **Group2** | **Group3** | **Group4** | **Group5** | **Group6** | **Kruskal Wallis test statistic** | | **Dunn-Bonferroni post hoc test differences** |
|  | **Item** | *18-24* | *25-34* | *35-44* | *45-54* | *55-64* | *>65* | $\chi{2\left( 5 \right)}$ | p | (p-value) |
| Q1 | Q1-A | 3,48 | 3,12 | 3,27 | 3,52 | 3,55 | 3,57 | 30,362 | <,001 | 2-1 (,006), 2-4 (,001), 2-5 (,001) |
|  | Q1-B | 3,71 | 3,38 | 3,47 | 3,74 | 3,84 | 3,73 | 23,020 | <,001 | 2-1 (,038), 2-4 (,018), 2-5 (,002) |
|  | Q1-C | 3,51 | 3,44 | 3,56 | 3,50 | 3,67 | 3,38 | 8,339 | ,138 |  |
|  | Q1-D | 3,76 | 3,57 | 3,65 | 3,86 | 3,99 | 3,97 | 28,764 | <,001 | 2-4 (,020), 2-5 (<,001), 5-1 (,021), 5-3 (,024) |
|  | Q1-E | 3,81 | 3,62 | 3,57 | 3,80 | 3,94 | 3,90 | 17,327 | ,004 | 5-2 (,006), 5-3 (,031) |
|  | Q1-F | 3,65 | 3,38 | 3,50 | 3,73 | 3,84 | 3,83 | 28,292 | <,001 | 2-4 (,003), 2-5 (<,001) |
| Q2 | Q2-A | 3,50 | 3,46 | 3,35 | 3,59 | 3,56 | 3,98 | 21,975 | ,001 | 6-1 (,008), 6-2 (,003), 6-3 (<,001) |
|  | Q2-B | 2,99 | 2,77 | 2,85 | 3,12 | 3,20 | 3,38 | 35,709 | <,001 | 2-4 (002), 2-5 (<,001), 2-6 (,002), 3-5 (,042), 3-6 (0,26) |
|  | Q2-C | 3,00 | 2,75 | 2,86 | 3,06 | 3,18 | 3,13 | 25,018 | <,001 | 2-4 (,021), 2-5 (<,001) |
|  | Q2-D | 2,90 | 2,69 | 2,80 | 3,05 | 3,19 | 3,18 | 35,170 | <,001 | 2-4 (,002), 2-5 (<,001), 2-6 (,037), 5-3 (,020) |
|  | Q2-E | 3,10 | 2,87 | 3,03 | 3,21 | 3,38 | 3,50 | 33,601 | <,001 | 2-4 (,009), 2-5 (<,001), 2-6 (,005) |
|  | Q2-F | 2,73 | 2,60 | 2,80 | 2,91 | 3,24 | 3,00 | 39,832 | <,001 | 5-1 (<,001), 5-2 (<,001), 5-3 (,007), 2-4 (,032) |
|  | Q2-G | 3,20 | 2,89 | 3,13 | 3,23 | 3,49 | 3,62 | 39,140 | <,001 | 2-1 (,042), 2-4 (,026), 2-5 (<,001), 2-6 (,002) |
| Q3 | Q3-A | 2,97 | 2,71 | 3,00 | 3,06 | 3,16 | 3,02 | 25,843 | <,001 | 2-4 (,006), 2-5 (<,001) |
|  | Q3-B | 2,99 | 2,82 | 3,08 | 3,09 | 3,21 | 3,00 | 19,797 | ,001 | 2-4 (,042), 2-5 (,002) |
|  | Q3-C | 3,18 | 3,06 | 3,16 | 3,26 | 3,33 | 3,23 | 9,846 | ,080 |  |
|  | Q3-D | 3,14 | 2,85 | 3,07 | 3,21 | 3,36 | 3,13 | 27,771 | <,001 | 2-4 (,005), 2-5 (<,001) |
|  | Q3-E | 3,10 | 2,91 | 3,06 | 3,11 | 3,26 | 3,23 | 15,571 | ,008 | 2-5 (,006) |
|  | Q3-F | 3,10 | 2,96 | 3,09 | 3,10 | 3,23 | 3,12 | 9,175 | ,102 |  |
|  | Q3-G | 2,89 | 2,68 | 2,87 | 2,97 | 3,06 | 2,97 | 19,093 | ,002 | 2-4 (,026), 2-5 (,003) |
| Q4 | Q4-A | 3,06 | 3,10 | 3,22 | 3,29 | 3,40 | 3,28 | 17,252 | ,004 | 5-1 (,008), 5-2 (,029) |
|  | Q4-B | 3,26 | 3,09 | 3,27 | 3,40 | 3,52 | 3,67 | 28,099 | <,001 | 2-4 (,022), 2-5 (,001), 2-6 (,009) |
|  | Q4-C | 3,43 | 3,34 | 3,41 | 3,60 | 3,67 | 3,63 | 18,459 | ,002 | 2-5 (,006) |
|  | Q4-D | 2,69 | 2,54 | 2,56 | 2,81 | 3,11 | 2,80 | 33,587 | <,001 | 5-1 (,007), 5-2 (<,001), 5-3 (<,001) |
|  | Q4-E | 2,68 | 2,55 | 2,73 | 2,94 | 3,06 | 2,82 | 30,761 | <,001 | 2-4 (,001), 2-5 (<,001), 5-1 (,011) |
|  | Q4-F | 2,93 | 2,59 | 2,73 | 2,93 | 3,09 | 3,00 | 34,091 | <,001 | 2-1 (,002), 2-4 (,007), 2-5 (<,001), 5-3 (,028) |
| Q5 | Q5-A | 3,05 | 3,11 | 3,17 | 3,19 | 3,30 | 3,23 | 8,552 | ,128 |  |
|  | Q5-B | 3,23 | 3,03 | 3,14 | 3,24 | 3,24 | 3,43 | 12,344 | ,030 |  |
|  | Q5-C | 3,31 | 3,19 | 3,21 | 3,31 | 3,38 | 3,43 | 7,619 | ,178 |  |
|  | Q5-D | 2,68 | 2,40 | 2,53 | 2,71 | 2,79 | 2,92 | 29,907 | <,001 | 2-1 (,010), 2-4 (,012), 2-5 (<,001), 2-6 (,016) |
|  | Q5-E | 2,62 | 2,48 | 2,63 | 2,81 | 2,88 | 2,98 | 27,531 | <,001 | 2-4 (,006), 2-5 (,001), 2-6 (,023) |
|  | Q5-F | 2,63 | 2,46 | 2,73 | 2,74 | 2,92 | 2,95 | 36,018 | <,001 | 2-3 (,023), 2-4 (,014), 2-5 (<,001), 2-6 (,010), 5-1 (,037) |
| Q6 | Q6-A | 3,47 | 3,46 | 3,55 | 3,65 | 3,59 | 3,35 | 10,547 | ,061 |  |
|  | Q6-B | 3,59 | 3,57 | 3,58 | 3,71 | 3,63 | 3,63 | 5,261 | ,385 |  |
|  | Q6-C | 3,62 | 3,67 | 3,68 | 3,76 | 3,67 | 3,60 | 5,480 | ,360 |  |
|  | Q6-D | 3,25 | 3,18 | 3,14 | 3,25 | 3,26 | 3,17 | 5,657 | ,341 |  |
|  | Q6-E | 3,24 | 3,24 | 3,33 | 3,32 | 3,32 | 3,27 | 5,121 | ,401 |  |
|  | Q6-F | 3,29 | 3,29 | 3,27 | 3,39 | 3,37 | 3,33 | 5,799 | ,326 |  |
| Q7 | Q7-A | 3,48 | 3,43 | 3,35 | 3,54 | 3,54 | 3,62 | 8,044 | ,154 |  |
|  | Q7-B | 3,88 | 3,80 | 3,76 | 3,55 | 3,34 | 3,35 | 52,862 | <,001 | 5-1 (<,001), 5-2 (<,001), 5-3 (,001), 6-1 (,007), 4-1 (,001), 4-2 (,034) |
|  | Q7-C | 2,98 | 2,90 | 2,74 | 2,77 | 2,63 | 2,70 | 30,559 | <,001 | 1-3 (,033), 1-4 (,013), 1-5 (<,001), 5-2 (,002) |
|  | Q7-D | 3,46 | 3,51 | 3,44 | 3,44 | 3,35 | 3,50 | 5,699 | ,337 |  |
|  | Q7-E | 3,33 | 3,27 | 3,30 | 3,35 | 3,33 | 3,45 | 5,065 | ,408 |  |
|  | Q7-F | 3,30 | 3,31 | 3,31 | 3,46 | 3,46 | 3,55 | 16,390 | ,006 |  |
| Q8 | Q8-A | 3,29 | 3,22 | 3,27 | 3,32 | 3,34 | 3,40 | 4,779 | ,443 |  |
|  | Q8-B | 3,39 | 3,28 | 3,27 | 3,30 | 3,30 | 3,38 | 8,636 | ,124 |  |
|  | Q8-C | 3,25 | 3,21 | 3,24 | 3,27 | 3,26 | 3,30 | 3,333 | ,649 |  |
|  | Q8-D | 3,29 | 3,26 | 3,21 | 3,33 | 3,33 | 3,37 | 3,866 | ,569 |  |
| Q9 | Q9-A | 3,08 | 3,09 | 3,01 | 3,09 | 3,15 | 3,12 | 3,061 | ,691 |  |
|  | Q9-B | 2,71 | 2,63 | 2,64 | 2,74 | 2,79 | 2,77 | 7,833 | ,166 |  |
|  | Q9-C | 2,22 | 2,30 | 2,46 | 2,50 | 2,40 | 2,52 | 18,923 | ,002 | 1-4 (,006) |
|  | Q9-D | 3,08 | 3,19 | 2,96 | 2,85 | 2,77 | 2,83 | 37,164 | <,001 | 2-4 (<,001), 2-5 (<,001), 5-1 (,006) |
|  | Q9-E | 3,64 | 3,54 | 3,50 | 3,64 | 3,53 | 3,60 | 7,844 | ,165 |  |
| Q10 | Q10-A | 3,46 | 3,39 | 3,33 | 3,40 | 3,44 | 3,52 | 8,517 | ,130 |  |
|  | Q10-B | 3,49 | 3,47 | 3,42 | 3,45 | 3,54 | 3,47 | 2,918 | ,713 |  |
|  | Q10-C | 3,24 | 3,13 | 3,14 | 3,15 | 3,24 | 3,18 | 10,757 | ,056 |  |
|  | Q10-D | 3,24 | 3,16 | 3,27 | 3,24 | 3,30 | 3,30 | 8,875 | ,114 |  |
|  | Q10-E | 3,27 | 3,16 | 3,13 | 3,15 | 3,27 | 3,37 | 14,401 | ,013 |  |

*The notation “x-y (,zzz)” in the Differences column indicates that groups x and y are significantly different with a p-value of 0,zzz.*

**Table A4 - Income comparison. Results of the Kruskal-Wallis test and Dunn-Bonferroni post hoc test.**

|  |  |  | **Means** |  |  | **Sign.** | | |
| --- | --- | --- | --- | --- | --- | --- | --- | --- |
| **Question** | | **Group 1** | **Group 2** | **Group 3** | **Group 4** | **Kruskal Wallis test statistic** | | **Dunn-Bonferroni post hoc test differences** |
|  | **Item** | < 1.500 | 1.500 - 3.000 | 3.001 - 4.500 | > 4.500 | $\chi{2\left( 3 \right)}$ | p | (p-value) |
| Q1 | Q1-A | 3,42 | 3,42 | 3,20 | 3,26 | 9,519 | ,023 | 3-2 (,033) |
|  | Q1-B | 3,69 | 3,64 | 3,50 | 3,39 | 6,764 | ,080 |  |
|  | Q1-C | 3,44 | 3,51 | 3,57 | 3,49 | 2,334 | ,506 |  |
|  | Q1-D | 3,73 | 3,75 | 3,75 | 3,66 | 0,286 | ,963 |  |
|  | Q1-E | 3,67 | 3,74 | 3,76 | 3,75 | 1,305 | ,728 |  |
|  | Q1-F | 3,49 | 3,62 | 3,65 | 3,46 | 4,530 | ,210 |  |
| Q2 | Q2-A | 3,50 | 3,51 | 3,49 | 3,52 | 0,471 | ,925 |  |
|  | Q2-B | 3,12 | 3,02 | 2,78 | 2,83 | 15,416 | ,001 | 3-1 (,006), 3-2 (,020) |
|  | Q2-C | 3,05 | 3,01 | 2,75 | 2,79 | 14,279 | ,003 | 3-1 (,027), 3-2 (,010) |
|  | Q2-D | 2,90 | 2,96 | 2,77 | 2,80 | 6,539 | ,088 |  |
|  | Q2-E | 3,19 | 3,18 | 2,86 | 2,90 | 20,067 | <,001 | 3-1 (,007), 3-2 (,001) |
|  | Q2-F | 2,96 | 2,87 | 2,58 | 2,66 | 16,839 | ,001 | 3-1 (,004), 3-2 (,006) |
|  | Q2-G | 3,35 | 3,25 | 2,89 | 2,88 | 29,448 | <,001 | 3-1 (<,001), 3-2 (<,001), 4-1 (,002), 4-2 (,008) |
| Q3 | Q3-A | 3,03 | 2,99 | 2,82 | 2,71 | 10,792 | ,013 |  |
|  | Q3-B | 2,96 | 3,04 | 2,98 | 2,84 | 4,206 | ,240 |  |
|  | Q3-C | 3,13 | 3,21 | 3,13 | 3,17 | 1,580 | ,664 |  |
|  | Q3-D | 3,21 | 3,12 | 2,97 | 2,91 | 8,143 | ,043 |  |
|  | Q3-E | 3,07 | 3,09 | 3,00 | 3,07 | 0,968 | ,809 |  |
|  | Q3-F | 3,02 | 3,09 | 3,07 | 3,07 | 0,789 | ,852 |  |
|  | Q3-G | 2,79 | 2,88 | 2,86 | 2,85 | 1,405 | ,704 |  |
| Q4 | Q4-A | 3,13 | 3,15 | 3,24 | 3,31 | 3,469 | ,325 |  |
|  | Q4-B | 3,38 | 3,27 | 3,18 | 3,37 | 5,166 | ,160 |  |
|  | Q4-C | 3,53 | 3,45 | 3,43 | 3,48 | 1,546 | ,672 |  |
|  | Q4-D | 2,80 | 2,76 | 2,57 | 2,53 | 10,754 | ,013 |  |
|  | Q4-E | 2,90 | 2,76 | 2,57 | 2,72 | 9,417 | ,024 | 1-3 (,017) |
|  | Q4-F | 2,87 | 2,87 | 2,70 | 2,76 | 5,006 | ,171 |  |
| Q5 | Q5-A | 3,15 | 3,15 | 3,13 | 3,17 | 0,189 | ,979 |  |
|  | Q5-B | 3,21 | 3,20 | 3,05 | 3,19 | 4,760 | ,190 |  |
|  | Q5-C | 3,31 | 3,28 | 3,24 | 3,23 | 1,377 | ,711 |  |
|  | Q5-D | 2,71 | 2,63 | 2,51 | 2,44 | 9,023 | ,029 |  |
|  | Q5-E | 2,76 | 2,69 | 2,55 | 2,52 | 7,142 | ,068 |  |
|  | Q5-F | 2,72 | 2,69 | 2,55 | 2,61 | 5,581 | ,134 |  |
| Q6 | Q6-A | 3,46 | 3,50 | 3,55 | 3,61 | 5,498 | ,139 |  |
|  | Q6-B | 3,57 | 3,62 | 3,57 | 3,67 | 2,115 | ,549 |  |
|  | Q6-C | 3,65 | 3,66 | 3,67 | 3,75 | 1,233 | ,745 |  |
|  | Q6-D | 3,28 | 3,20 | 3,18 | 3,22 | 7,173 | ,067 |  |
|  | Q6-E | 3,36 | 3,28 | 3,20 | 3,28 | 9,339 | ,025 | 1-3 (,018) |
|  | Q6-F | 3,37 | 3,3 | 3,26 | 3,37 | 6,868 | ,076 |  |
| Q7 | Q7-A | 3,41 | 3,48 | 3,43 | 3,58 | 4,489 | ,213 |  |
|  | Q7-B | 3,54 | 3,64 | 3,84 | 3,90 | 21,980 | <,001 | 1-3 (,004), 1-4 (,001), 2-3 (,022), 2-4 (,008) |
|  | Q7-C | 2,90 | 2,83 | 2,75 | 2,89 | 5,986 | ,112 |  |
|  | Q7-D | 3,48 | 3,48 | 3,38 | 3,44 | 2,896 | ,408 |  |
|  | Q7-E | 3,40 | 3,31 | 3,23 | 3,36 | 7,150 | ,067 |  |
|  | Q7-F | 3,44 | 3,36 | 3,27 | 3,40 | 7,483 | ,058 |  |
| Q8 | Q8-A | 3,40 | 3,26 | 3,30 | 3,14 | 13,011 | ,005 | 1-4 (,004) |
|  | Q8-B | 3,34 | 3,35 | 3,26 | 3,21 | 10,569 | ,014 | 2-4 (,030) |
|  | Q8-C | 3,24 | 3,26 | 3,17 | 3,28 | 5,477 | ,140 |  |
|  | Q8-D | 3,33 | 3,26 | 3,27 | 3,33 | 2,714 | ,438 |  |
| Q9 | Q9-A | 2,96 | 3,06 | 3,18 | 3,23 | 11,766 | ,008 | 1-3 (,046), 1-4 (,049) |
|  | Q9-B | 2,59 | 2,65 | 2,81 | 2,79 | 10,769 | ,013 | 1-3 (,027) |
|  | Q9-C | 2,41 | 2,41 | 2,25 | 2,23 | 8,711 | ,033 |  |
|  | Q9-D | 3,09 | 2,96 | 3,01 | 3,15 | 5,892 | ,117 |  |
|  | Q9-E | 3,53 | 3,55 | 3,62 | 3,67 | 4,096 | ,251 |  |
| Q10 | Q10-A | 3,42 | 3,41 | 3,36 | 3,46 | 2,565 | ,464 |  |
|  | Q10-B | 3,40 | 3,47 | 3,47 | 3,59 | 7,156 | ,067 |  |
|  | Q10-C | 3,19 | 3,17 | 3,15 | 3,24 | 1,628 | ,653 |  |
|  | Q10-D | 3,25 | 3,23 | 3,16 | 3,31 | 5,553 | ,136 |  |
|  | Q10-E | 3,16 | 3,21 | 3,17 | 3,29 | 5,417 | ,144 |  |

*The notation “x-y (,zzz)” in the “Dunn-Bonferroni post hoc test differences” column indicates that groups x and y are significantly different with a p-value of 0,zzz.*

**Table A5 - Education comparison. Results of the Kruskal-Wallis test and Dunn-Bonferroni post hoc test.**

| **Means** | | | | | | **Sign.** | | |
| --- | --- | --- | --- | --- | --- | --- | --- | --- |
| **Question** | | **Group**  **1** | **Group**  **2** | **Group**  **3** | **Group**  **4** | **Kruskal Wallis test statistic** | | **Dunn-Bonferroni post hoc test differences** |
|  | **Item** | *Middle School or lower* | *High school* | *Bachelor’s or master’s degree* | *Postgraduate course or PhD* | $\chi{2\left( 3 \right)}$ | p | (p-value) |
| Q1 | Q1-A | 3,6 | 3,52 | 3,27 | 3,08 | 22,339 | <,001 | 4-1 (,037), 4-2 (<,001), 2-3 (,003) |
|  | Q1-B | 3,94 | 3,80 | 3,50 | 3,16 | 36,539 | <,001 | 4-1 (<,001), 4-2 (<,001), 4-3 (,033), 3-1 (,039), 3-2 (,001) |
|  | Q1-C | 3,08 | 3,48 | 3,60 | 3,45 | 20,146 | <,001 | 1-2 (,016), 1-3 (<,001), 1-4 (,027) |
|  | Q1-D | 3,52 | 3,85 | 3,71 | 3,57 | 9,409 | ,024 |  |
|  | Q1-E | 3,6 | 3,80 | 3,73 | 3,64 | 5,082 | ,166 |  |
|  | Q1-F | 3,51 | 3,66 | 3,57 | 3,46 | 2,803 | ,423 |  |
| Q2 | Q2-A | 3,54 | 3,53 | 3,52 | 3,37 | 4,300 | ,231 |  |
|  | Q2-B | 3,13 | 3,15 | 2,86 | 2,70 | 29,446 | <,001 | 2-3 (<,001), 2-4 (<,001) |
|  | Q2-C | 3,23 | 3,09 | 2,85 | 2,72 | 22,850 | <,001 | 1-4 (,016), 2-3 (,002), 2-4 (,002) |
|  | Q2-D | 2,9 | 3,02 | 2,83 | 2,72 | 11,089 | ,011 | 2-3 (,037), 2-4 (,035) |
|  | Q2-E | 3,3 | 3,29 | 2,98 | 2,77 | 31,677 | <,001 | 4-1 (,012), 4-2 (<,001), 2-3 (<,001) |
|  | Q2-F | 2,95 | 2,98 | 2,69 | 2,62 | 21,481 | <,001 | 2-3 (<,001), 2-4 (,006) |
|  | Q2-G | 3,54 | 3,32 | 3,05 | 2,86 | 27,487 | <,001 | 1-3 (,012), 1-4 (,001), 2-3 (,002), 2-4 (,001) |
| Q3 | Q3-A | 2,98 | 3,08 | 2,82 | 2,86 | 12,437 | ,006 | 2-3 (,003) |
|  | Q3-B | 2,79 | 3,10 | 2,93 | 2,98 | 8,600 | ,035 |  |
|  | Q3-C | 3,11 | 3,31 | 3,09 | 3,11 | 10,914 | ,012 | 2-3 (,011) |
|  | Q3-D | 3,08 | 3,28 | 2,98 | 2,86 | 21,593 | <,001 | 2-3 (<,001), 2-4 (,001) |
|  | Q3-E | 3,02 | 3,14 | 3,03 | 2,99 | 3,066 | ,382 |  |
|  | Q3-F | 2,95 | 3,11 | 3,05 | 3,10 | 2,061 | ,560 |  |
|  | Q3-G | 2,74 | 2,91 | 2,84 | 2,82 | 2,410 | ,492 |  |
| Q4 | Q4-A | 3,21 | 3,24 | 3,13 | 3,21 | 2,366 | ,500 |  |
|  | Q4-B | 3,51 | 3,39 | 3,20 | 3,13 | 11,556 | ,009 | 2-3 (,045) |
|  | Q4-C | 3,71 | 3,52 | 3,42 | 3,32 | 7,521 | ,057 |  |
|  | Q4-D | 2,93 | 2,80 | 2,65 | 2,48 | 14,019 | ,003 | 4-1 (,023), 4-2 (,012) |
|  | Q4-E | 2,80 | 2,88 | 2,67 | 2,56 | 12,107 | ,007 | 2-3 (,030), 2-4 (,021) |
|  | Q4-F | 2,98 | 2,97 | 2,72 | 2,68 | 15,604 | ,001 | 2-3 (,004), 2-4 (,035) |
| Q5 | Q5-A | 3,25 | 3,19 | 3,09 | 3,22 | 3,486 | ,323 |  |
|  | Q5-B | 3,44 | 3,25 | 3,10 | 3,05 | 10,154 | ,017 |  |
|  | Q5-C | 3,51 | 3,34 | 3,20 | 3,22 | 7,893 | ,048 |  |
|  | Q5-D | 3,04 | 2,73 | 2,50 | 2,39 | 30,952 | <,001 | 1-3 (<,001), 1-4 (<,001), 2-3 (,002), 2-4 (,003) |
|  | Q5-E | 2,77 | 2,77 | 2,58 | 2,52 | 11,329 | ,010 | 2-3 (,031) |
|  | Q5-F | 2,75 | 2,78 | 2,56 | 2,59 | 12,237 | ,007 | 2-3 (,005) |
| Q6 | Q6-A | 3,39 | 3,51 | 3,50 | 3,64 | 7,563 | ,056 |  |
|  | Q6-B | 3,65 | 3,63 | 3,59 | 3,58 | 1,493 | ,684 |  |
|  | Q6-C | 3,61 | 3,65 | 3,68 | 3,74 | 2,752 | ,431 |  |
|  | Q6-D | 3,2 | 3,23 | 3,21 | 3,15 | 4,159 | ,245 |  |
|  | Q6-E | 3,23 | 3,27 | 3,29 | 3,27 | 1,322 | ,724 |  |
|  | Q6-F | 3,27 | 3,31 | 3,32 | 3,31 | 0,417 | 0,937 |  |
| Q7 | Q7-A | 3,39 | 3,51 | 3,44 | 3,48 | 4,083 | ,253 |  |
|  | Q7-B | 3,31 | 3,64 | 3,75 | 3,85 | 17,942 | <,001 | 1-2 (,034), 1-3 (,002), 1-4 (,001) |
|  | Q7-C | 2,76 | 2,85 | 2,85 | 2,76 | 3,561 | ,313 |  |
|  | Q7-D | 3,33 | 3,48 | 3,47 | 3,37 | 3,882 | ,274 |  |
|  | Q7-E | 3,39 | 3,38 | 3,29 | 3,21 | 10,187 | ,017 | 2-4 (,015) |
|  | Q7-F | 3,5 | 3,39 | 3,34 | 3,28 | 6,202 | ,102 |  |
| Q8 | Q8-A | 3,07 | 3,33 | 3,27 | 3,26 | 7,169 | ,067 |  |
|  | Q8-B | 3,26 | 3,37 | 3,29 | 3,22 | 8,240 | ,041 |  |
|  | Q8-C | 3,12 | 3,27 | 3,25 | 3,20 | 3,726 | ,293 |  |
|  | Q8-D | 3,08 | 3,28 | 3,30 | 3,29 | 7,505 | ,057 |  |
| Q9 | Q9-A | 2,99 | 3,02 | 3,12 | 3,18 | 6,051 | ,109 |  |
|  | Q9-B | 2,42 | 2,67 | 2,72 | 2,74 | 12,335 | ,006 | 1-2 (,018), 1-3 (,004), 1-4 (,008) |
|  | Q9-C | 2,44 | 2,38 | 2,33 | 2,36 | 1,538 | ,673 |  |
|  | Q9-D | 2,99 | 2,96 | 3,02 | 3,15 | 5,326 | ,149 |  |
|  | Q9-E | 3,49 | 3,51 | 3,61 | 3,69 | 7,450 | ,059 |  |
| Q10 | Q10-A | 3,32 | 3,42 | 3,41 | 3,42 | 0,512 | ,916 |  |
|  | Q10-B | 3,38 | 3,43 | 3,50 | 3,56 | 8,562 | ,036 |  |
|  | Q10-C | 3,23 | 3,17 | 3,18 | 3,16 | 0,711 | ,871 |  |
|  | Q10-D | 3,13 | 3,23 | 3,24 | 3,23 | 2,100 | ,552 |  |
|  | Q10-E | 3,05 | 3,17 | 3,23 | 3,25 | 6,933 | ,074 |  |

*The notation “x-y (,zzz)” in the Differences column indicates that groups x and y are significantly different with a p-value of 0,zzz.*
